# Development of a program for *in silico* optimized selection of oligonucleotide-based molecular barcodes

**DOI:** 10.1371/journal.pone.0246354

**Published:** 2021-02-18

**Authors:** In Seok Yang, Sang Won Bae, BeumJin Park, Sangwoo Kim

**Affiliations:** 1 Department of Biomedical Systems Informatics and Brain Korea 21 PLUS Project for Medical Science, Yonsei University College of Medicine, Seoul, Korea; 2 Department of Computer Science, Kyonggi University, Suwon, Korea; University of Helsinki, FINLAND

## Abstract

Short DNA oligonucleotides (~4 mer) have been used to index samples from different sources, such as in multiplex sequencing. Presently, longer oligonucleotides (8–12 mer) are being used as molecular barcodes with which to distinguish among raw DNA molecules in many high-tech sequence analyses, including low-frequent mutation detection, quantitative transcriptome analysis, and single-cell sequencing. Despite some advantages of using molecular barcodes with random sequences, such an approach, however, makes it impossible to know the exact sequences used in an experiment and can lead to inaccurate interpretation due to misclustering of barcodes arising from the occurrence of unexpected mutations in the barcodes. The present study introduces a tool developed for selecting an optimal barcode subset during molecular barcoding. The program considers five barcode factors: GC content, homopolymers, simple sequence repeats with repeated units of dinucleotides, Hamming distance, and complementarity between barcodes. To evaluate a selected barcode set, penalty scores for the factors are defined based on their distributions observed in random barcodes. The algorithm employed in the program comprises two steps: i) random generation of an initial set and ii) optimal barcode selection via iterative replacement. Users can execute the program by inputting barcode length and the number of barcodes to be generated. Furthermore, the program accepts a user’s own values for other parameters, including penalty scores, for advanced use, allowing it to be applied in various conditions. In many test runs to obtain 100000 barcodes with lengths of 12 nucleotides, the program showed fast performance, efficient enough to generate optimal barcode sequences with merely the use of a desktop PC. We also showed that VFOS has comparable performance, flexibility in program running, consideration of simple sequence repeats, and fast computation time in comparison with other two tools (DNABarcodes and FreeBarcodes). Owing to the versatility and fast performance of the program, we expect that many researchers will opt to apply it for selecting optimal barcode sets during their experiments, including next-generation sequencing.

## Introduction

DNA barcodes are oligonucleotide sequences tagged to target DNA molecules that allow researchers to identify specific molecules in an experiment, including sequencing experiments [1, 2]. There are two general types of DNA barcodes [3]: The first are DNA barcodes that permit the identification of individual samples in a pooled mixture. For the purpose, short DNA barcodes (~4 mer oligonucleotides) are frequently used. The second are molecular barcodes, also known as unique molecular identifiers, that allow for consensus-based error correction by facilitating the unique labeling of individual molecules [4]. In many high-tech sequence analyses, longer barcodes (8–12 mer) of this second type are used to identify raw DNA molecules. DNA barcodes can also be characterized according to their design (i.e., rationally designed or randomly produced) [5], and random barcodes are often used for molecular barcoding [2, 6–8]: note that the barcodes mentioned in this study indicate “in-line barcodes” to be sequenced together with target DNA sequences.

Next-generation sequencing (NGS) has been widely applied in genomic and transcriptomic analyses for various purposes [9], including clinical research [10, 11]. In NGS data analysis, the read depth (i.e., number of reads) of a target region has been used to identify variant alleles and the frequencies thereof [12] and to estimate expression levels of genes [13]. However, this approach sometimes suffers from amplification bias between samples during library preparation and errors in the sequencing step, including incorrect base incorporation. To overcome these issues, researchers have turned to tagging barcodes to individual DNA or RNA fragments, otherwise known as molecular barcoding, in an attempt to reduce amplification bias and to eliminate false positive variants by filtering out duplicate reads. This approach has been applied in various ways: One, it has been used in many clinical studies to identify true variants with very low allele frequencies less than or equal to 1% in liquid biopsies from cancers [14]. Two, it has been implemented in quantitative transcriptome analysis to allow for more accurate quantification of transcript levels [15]. Three, it has also been applied in single-cell sequencing of large cell populations, wherein micro-fluidic droplet barcoding was used to label the genomes of single cells in each droplet [16, 17].

For research purposes, barcodes for random sequences have been used [2, 6–8], which is reasonable because any number of barcodes can be readily produced. However, this makes it impossible to know the exact sequences of all barcodes used in an experiment, thereby hindering a researcher’s ability to identify which base(s) in a barcode was/were mutated. Furthermore, misclustering of barcodes in data analysis due to unexpected mutations in their sequences can sometimes lead to inaccurate interpretation in the identification of mutations with low allele frequency, in the quantification of transcript-level expressions in disease samples, and even in single-cell sequencing. Therefore, we presumed that the preselection of an optimal barcode set in sequencing experiments would considerably reduce the occurrences of the above.

To date, several studies have addressed DNA barcode design [18–23], focusing on detection of mismatches (substitution, insertion, and deletion) between expected (original) and observed barcodes, as well as the correction of such errors and the removal of duplicated reads (i.e., demultiplexing). For barcode design, researchers have considered the length of barcodes, the minimum distance (MD) between two barcodes, GC content (GCC), homopolymers (HPs), and complementarity (CP) between barcodes. For example, balanced GCC (e.g., 40–60%), HPs of minimal length (e.g., <3), and/or minimum CPs (e.g., <3) have been utilized to filter out undesirable barcodes in the generation step. Furthermore, since mutations can be occurred in-line barcodes due to the amplification with primers during library preparation and sequencing together with target DNA molecules during sequencing reactions, mismatched bases between original and mutated ones (i.e., *k* errors) should be considered in the barcode design step. For detecting and correcting *k* errors, MD values in barcode sets have been controlled at greater than or equal to *k*+1 and 2*k*+1, respectively, calculated based on distance metrics such as Hamming and Levenshtein distances (HD and LD, respectively). Meanwhile, however, researchers have yet to account for simple sequence repeats with repeated units of dinucleotides (SRs), which are somewhat similar to HPs and are frequently observed in longer barcodes (e.g., 8–12 mer). In this reason, SR is another possible source of errors in PCR amplification and sequencing steps as previously reported [24].

The barcode factors of lengths, GCC, HP, SR, and CP have been commonly considered in PCR primer design [25]. For example, primers with unbalanced GCCs can lead to mispriming and misannealing during PCRs [26]. Also, the presence of long HPs or SRs in the primers can cause polymerase slippage [27], thereby yielding insertion or deletion in the regions [28]. Sometimes, two primers complementing each other can form a dimer during PCRs, reducing the product yield [25]. Finally, platform-specific errors occur in NGS, especially in certain genome regions with GC-rich, long HPs, and repeat sequences [29, 30].

Here, we present a versatile and fast program (VFOS) that is capable of selecting an optimal subset of oligonucleotide sequences (barcodes) by considering penalty scores for five barcode factors, along with their relative importance. The program allows a potential user to adjust penalty scores and weights to yield barcode sets that best fit their needs. Therefore, we expect that many researchers will choose VFOS to obtain their own barcode sets that can be applied to various experimental conditions.

## Methods

The VFOS program was developed by implementing C++ language, and the g++ compiler was used for its compilation from source code on a Linux operating system. The detailed methods for selecting optimal barcode sequences with VFOS are described below.

### Definition of a barcode sequence

A barcode is an oligonucleotide sequence of four bases {A, T, G, C}. We define *l* as the length of a barcode. For given *l*, there are 4^*l*^ possible barcodes. For example, a total of 1048576 and 16777216 unique barcodes can be generated for *l* = 10 and 12, respectively.

### Definition of barcode factors

We defined five barcode factors (GCC, HP, SR, HD, and CP) that are considered in selecting an optimal barcode subset. *gcc* is the percentage of nitrogenous bases (guanine [G] and cytosine [C]) in a barcode, calculated as follows: $gcc(B)=\frac{n_{G}(B)+n_{C}(B)}{l(B)}\times 100$, where *n*_*G*_(*B*) and *n*_*C*_(*B*) are the number of guanines and cytosines in *B*, respectively. For example, *gcc* of the sequence “AGCTAAGCTACC” is 50% (= 6/12). *hp* is defined as the length of the longest running single base repeats. For example, the *hp* of the sequence “GTAAACGGGGGC” is five. *sr* is similar to *hp*, but defined for dinucleotide repeats (e.g., AGAGAG or TCTCTCTC). In calculating *sr*, single base repeats are also considered, but as the repeat of dinucleotides (e.g., “GGGGGG”is a 3×“GG”repeat). *hd* and *cp* are defined for barcode pairs. *hd* is HD-based edit distance (the number of positions at which the corresponding bases are different in the ungapped pairwise alignment) between two barcodes. For example, *hd* of {“AAAAC”, “AGAAG”} is two. CP measures the degree of base pairing (“A-T” or “G-C”) between two barcodes, in the formation of self- or cross-dimers. For given two barcodes *B*_*1*_ and *B*_*2*_, *cp* is defined as the maximum number of Watson-Crick base pairs that is determined by sliding *B*_*1*_ on the *B*_*2*_ or vice versa with minimum overlap of *l* = 3 (see S1 Fig)_._ For example, there can be two sequences (“AGACAT” and “GTGTCC”) in relationship of *cp* = 4, because “GACA” in the first one and “TGTC” in the second one are reverse complementary. Note that if directionality of DNA or RNA sequences is omitted, the left and right ends of the sequences indicate 5′- and 3′-ends, respectively. Accordingly, “AGACAT” and “GTGTCC” mean “5′-AGACAT-3′” and “5′-GTGTCC-3′,” respectively.

From the definitions, we can deduce the desired conditions of the five factors. In general, extremely biased *gcc* towards 0% or 100% is avoided in the design of PCR primers [25], and can be applied to the barcode design. *hp*, *sr*, and *cp* should remain low to prevent unwanted errors (e.g., replication slippage or formation of barcode dimers). In addition, *hd* should be maximized for better discrimination between barcodes and tolerance from nucleotide variations. As these factors are considered in up to millions of barcodes, simultaneously, appropriate scoring with weight is the key to the optimization of the final set.

### Generation of random sequences to characterize DNA barcodes

To examine the characteristics of the five barcode factors, we generated random barcodes for three different lengths (*l* = 8, 10, and 12): these were chosen based on their frequency of use. For each *l*, we generated 1000 random barcode sets, each of which consists of 10^5^ (*l* = 10 and 12) or 10^4^ barcodes (*l* = 8).

### Determination of penalty scores for evaluating individual barcodes

To apply the barcode factors to the barcode set optimization, we used a penalty-based selection strategy. To determine the most appropriate penalty scores for GCC, HP, and SR (P_GCC_, P_HP_, and P_SR_, respectively), we obtained appropriate equations using the curve fitting method on the website MyCurveFit [31] based on distributions observed in random barcodes with *l* = 12. To obtain *gcc* distribution, we calculated mean barcode counts at each *gcc* value. A Gaussian bell curve with the formula $f(x)=a\;\times e^{-{\left(x-b\right)}^{2}/2c^{2}}$ was used to fit its distribution, where *x* indicates *gcc*. We set *f*(*gcc*) to be maximum if *gcc* was in the range from 40% to 60% and minimum at the points *gcc* = 0% or 100%. Then, we determined P_GCC_ values with a maximum value of 10^6^ at the points *gcc* = 0% or 100% and a minimum value of 0 in the range of *gcc* = 40% to 60% by inverting the distribution of *f*(*gcc*) values. For distributions of *hp* and *sr*, we adopted median barcode counts at each data point of *hp* and *sr*, respectively. When *hp* and *sr* were examined in a barcode sequence, we only considered them greater than or equal to 2, because there are many cases with *hp* = 1 and *sr* = 1 in a barcode. An exponential curve obtained with the formula *f*(*x*) = *a* + *b* × *e*^*cx*^ was applied to their random distributions, where *x* represents *hp* or *sr*. In the equations, constant terms (*a*) were removed to obtain *f*(*hp*) or *f*(*sr*) values greater than or equal to 0. We determined P_HP_ and P_SR_ scores by subtracting *f*(*hp*) and *f*(*sr*) values from 10^6^, respectively, with a modification to transform penalty scores into positive integers.

To generate penalty scores for HD (P_HD_), we implemented the concept of accumulation of mutations (herein, substitutions of a base at a nucleotide position) to represent distances or differences between barcode sequences. In a given barcode set with *l* = *n*, there exist barcode pairs with a relationship of *hd* values ranging from 1 to *n*. Since barcode sequences are sequenced with sample DNA or RNA fragments, unexpected mutations can be observed in the sequences. In result, barcodes with *hd* = 1, 2, or 3 are more sensitive to misclustering than others, sometimes leading to inaccurate interpretation in data analysis. Accordingly, we only dealt with P_HD_ values for *hd* = 1, 2, and 3 in this study. Since mutations can occur during library preparation or a sequencing step, rates for two error types (those for polymerases and sequencing platforms) warrant consideration. The former is known to range from 1/10^6^ to 1/10^5^ [32] and the latter approximately 1/10^3^ with the Illumina sequencing platform [29]. Of these, we selected the latter, because it is one thousand times higher than that of the former. Accordingly, if the probability that a mutation is detected in a sequence is *m* (= 1/10^3^), the probabilities that two and three mutations simultaneously occur would be *m*^2^ (= 1/10^6^) and *m*^3^ (= 1/10^9^), respectively. In this context, we defined P_HD_ values of barcodes with *hd* = 1, 2, and 3 as 10^6^, 10^3^, and 1, respectively, by dividing the probabilities by 1/10^9^. In addition, we set P_HD_ scores for barcodes with *hd*≥4 to 0, since the values were smaller than 1 and closer to 0 in the *hd* range (for example, 1/10^3^ and 1/10^6^ for those with *hd* = 4 and *hd* = 5, respectively). In addition, three look-up-tables containing precalculated *hd* values for short subsequences with *l* = 2, 3, and 4 are used to reduce computation time in HD calculation. For example, a barcode pair with *l* = 8 is composed of two pairs of subsequences with *l* = 4. Another pair with *l* = 9 is composed of three pairs of subsequences with *l* = 2, 3, and 4. Another pair *l* = 10 is composed of two pairs of subsequences with *l* = 4 and a pair of subsequences with *l* = 2. So, the total *hd* value of the pair can be obtained by summing *hd* values of subsequence pairs referred from look-up-tables. Note that longer subsequences are preferentially considered than shorter subsequences when referring to *hd* values from the look-up-tables.

To define penalty scores of CP, we employed maximum Watson-Crick base pairs between given barcodes rather than predicted free energies that has been widely used to predict DNA cross-hybridizations [33], because we had to consider computational time of free energies for all combinations of them. We also assumed that the strength of CP would increase dramatically if the *cp* between them became greater than a certain limit. Otherwise, it would become close or equal to its minimum value. We set the limit to 2/3 of *l*. The assumption aims to prevent inclusion of barcodes in relationships of full reverse complementary or close to it. For barcodes with *l* = 8, 10, and 12, the limits become 5.3, 7.5, and 9, respectively. The maximum value of P_CP_ score was set to 10^8^ for the barcodes in full complementary relationship. We also determined that the score will decrease by 10^2^ when *cp* decreases by 1. Thus, P_CP_ score was decreased to 1 at *cp* = 8. To reflect the nature of P_CP_, we chose an exponential model with the formula *f*(*x*) = *a* + *b* × *e*^*cx*^. In the equation, constant terms (*a*) were removed to obtain a *f*(*x*) value greater than or equal to 0.

Unlike penalty scores for GCC, HP, and SR, those for HD and CP are obtained by pairwise comparison between two barcodes and, thus, demand a greater number of calculations. To reduce calculation times for P_HP_ and P_CP_ for a barcode pair, we used look-up-tables containing pre-calculated *hd* values for two barcodes with *l* = 2, 3, and 4, respectively, as described above. Nonetheless, a total of $\frac{{\text{N}}^{2}}{2}-\text{N}$ calculations is required to obtain them for all barcode pairs, where N represents the total number of barcodes.

### Algorithm for selecting optimal barcode sets

In the VFOS program, we employed a simple algorithm consisting of two steps: random generation of an initial barcode set and optimal barcode selection via iterative replacement of barcodes. In every cycle of the second step, i) calculation of penalty scores for the selected barcode set, ii) determination of excluded barcodes based on a α value, and iii) addition of new barcodes are repeated. During the process, a weighted total penalty score (P_WT_) is applied to check whether a selected barcode set in each cycle is optimal and the best choice, which is calculated by summing all weighted penalty scores for the five barcode factors as shown in Eq (1)

$${\text{P}}_{\text{WT}}={\text{w}}_{1}\times {\text{P}}_{\text{GCCt}}+{\text{w}}_{2}\times {\text{P}}_{\text{HPt}}+{\text{w}}_{3}\times {\text{P}}_{\text{SRt}}+{\text{w}}_{4}\times {\text{P}}_{\text{HDt}}+{\text{w}}_{5}\times {\text{P}}_{\text{CPt}}$$ (1)

In the equation, P_GCCt_, P_HPt_, P_SRt_, P_HDt_, and P_CPt_ indicate total penalty scores for individual barcode factors in a barcode set as follows: ${\text{P}}_{\text{GCCt}}=\sum_{i=1}^{\text{N}}{\text{P}}_{\text{GCC}i}$, ${\text{P}}_{\text{HPt}}=\sum_{i=1}^{\text{N}}{\text{P}}_{\text{HP}i}$, ${\text{P}}_{\text{SRt}}=\sum_{i=1}^{\text{N}}{\text{P}}_{\text{SR}i}$, ${\text{P}}_{\text{HDt}}=\sum_{i=1}^{\text{N}}{\text{P}}_{\text{HD}i}$, and ${\text{P}}_{\text{CPt}}=\sum_{i=1}^{\text{N}}{\text{P}}_{\text{CP}i}$, where *i* stands for each barcode and N represents the total number of barcodes. This algorithm is stopped if P_WT_ score reaches its minimum limits through further running of an additional five cycles.

### Determination of weights for penalty scores and an appropriate α value

We first employed percent decrease (P_DEC_) to show the change of penalty score between initial and optimized states, which was calculated using the equation "(median P_a_ − median P_b_)/median P_a_ × 100" for each run, where P_a_ and P_b_ indicate initial and the lowest values for any penalty scores including P_WT_, respectively. Then, to find what combination of weights lead to the highest P_DEC_ values in a given space, we examined the values on the five dimensional space corresponding to weights (w_1_, w_2_, w_3_, w_4_, and w_5_) for five barcode factors (GCC, HP, SR, HD, and CP). Because P_DEC_ values at all data points could not be calculated due to the limitation of resource and time for computation, we chose only four points (1, 5, 10, and 20, respectively) per axis to consider total 4^5^ (= 1024) points on the space. Initial weights were set to values at the origin (w_1_ = 1, w_2_ = 1, w_3_ = 1, w_4_ = 1, and w_5_ = 1). By comparing all P_DEC_ values, final weights were determined to weight values at the point having the highest P_DEC_ value.

An appropriate α value was determined to that from a condition showing the minimum P_WT_ value at nearly 100 data points including the origin after running the VFOS program in six different conditions with α values of 5%, 10%, 20%, 30%, 40%, and 50%.

### Exclusion of barcodes in each cycle

The probability of barcode exclusion (P_EX_) is determined as shown in Eq (2), which is composed of the total penalty score for a barcode (P_T*i*_ = w_1_ × P_GCC*i*_ + w_2_ × P_HP*i*_ + w_3_ × P_SR*i*_ + w_4_ × P_HD*i*_ + w_5_ × P_CP*i*_), P_WT_, N, and α.

$${\text{P}}_{\text{EX}}=\frac{{\text{P}}_{\text{T}i}}{{\text{P}}_{\text{WT}}}\times \text{N}\times \alpha$$ (2)

A single barcode factor or a certain combination of the factors can be used as a monitoring target(s) during a program run by providing a user’s own penalty scores and weights. As all barcodes can be excluded with their own probabilities, depending on penalty scores, the algorithm used in the VFOS program avoids instances in which a barcode set falls into a local minimum depending on an initial state.

### Performance of the VFOS program

We tested the performance of VFOS in several conditions in which four cases were selected as representative examples: i) all barcode factors (GCC, HP, SR, HD, and CP) were considered; ii) only barcode factors GCC and HD were included; iii) only barcode factor GCC was examined; and iv) only barcode factor HD was considered in the test. The first condition was tested as a default setting of the VFOS program, and the remaining three conditions were examined as examples of user-defined settings. All results were obtained through 1000 repetitions of each condition.

### Comparison with other tools

For comparison of barcode sets from VFOS and two other tools (DNABarcodes [22] and FreeBarcodes [23]) that were designed to provide error-correcting barcode sets, we first created four barcode sets containing barcodes with *l* = 12 using DNABarcodes with minimum distances of 3 and 5, and FreeBarcodes with number of errors of 1 and 2. And barcodes with the same conditions for length and number that created under the respective settings of the two tools were also generated through 1000 repetitions using VFOS. Then, we compared the barcode sets with 6 criteria comprising five barcode factors (GCC, HP, SR, HD, and CP) and an additional factor (LD) as well as computation time between VFOS and other tools.

## Results

### Characteristics of random barcodes

We obtained distributions of random barcodes for five barcode factors according to *l*. S1–S3 Figs show the distributions for barcodes with *l* = 12. *gcc* distribution was well fitted to binomial distribution and centered on a *gcc* of 50% as expected (A). In *hp* distribution, the number of barcodes containing *hp* values for each base was nearly identical (B). Furthermore, HPs with *hp* = 2 were most frequently detected for all bases, and the number of HPs with *hp*>2 was drastically decreased with increasing *hp* (C). Note that only the *hp* distribution of an “A” base was represented due to a lack of space and very similar distributions between “A” and other bases (“T”, “G”, and “C”). The distribution of *sr* was similar with that for *hp*, in which SRs with *sr* = 2 were most frequently observed (D). *hd* distribution followed a right skewed pattern (E). When examining mode *hd* values according to *l*, approximately 2/3rds of bases for each *l* appeared (*hd* = 6, 8, and 9 for barcodes with *l* = 8, 10, and 12, respectively; S1–S3 Figs). *cp* distribution followed a different pattern than HD distribution (F), in which mode values were observed at approximately 1/3rds of bases for each *l* (*cp* = 3, 4, and 4 for barcodes with *l* = 8, 10, and 12, respectively; S1–S3 Figs).

### GCC penalty score (P_GCC_)

By fitting random *gcc* distribution to a Gaussian bell curve, we obtained an equation (Eq (3)) wherein μ = 50 and σ = 14.74996 (S5a Fig).

$$f(gcc)=22662.54\;\times \;e^{-\frac{{\left(gcc\;-50\right)}^{2}}{2\;\times \;14.74996^{2}}}$$ (3)

Then, we defined normalized *gcc* score, $\bar{gcc}$, as represented by Eq (4), to reflect that the best *gcc* range is from 40% to 60% in primer design for polymerase chain reaction and to set maximum $\bar{gcc}$ to 10^6^.

$$\bar{gcc}=\frac{f(gcc)}{f(gcc=40\;\text{or}\;60)}\times 10^{6}$$ (4)

In $\bar{gcc}$ distribution, $\bar{gcc}$ values ranged from 4023 (for the barcodes with *gcc* = 0% or 100%) to 10^6^ (for the barcodes with *gcc* values from 40% to 60%). Finally, we determined P_GCC_ values using Eq (5), where the minimum value of $\bar{gcc}$ (min($\bar{gcc}$)) represents $\bar{gcc}$ at *gcc* = 0% or 100%.

$${\text{P}}_{\text{GCC}}=\frac{10^{6}-\bar{gcc}}{10^{6}-\text{min}(\bar{gcc})}\times 10^{6}$$ (5)

**Fig 1:**
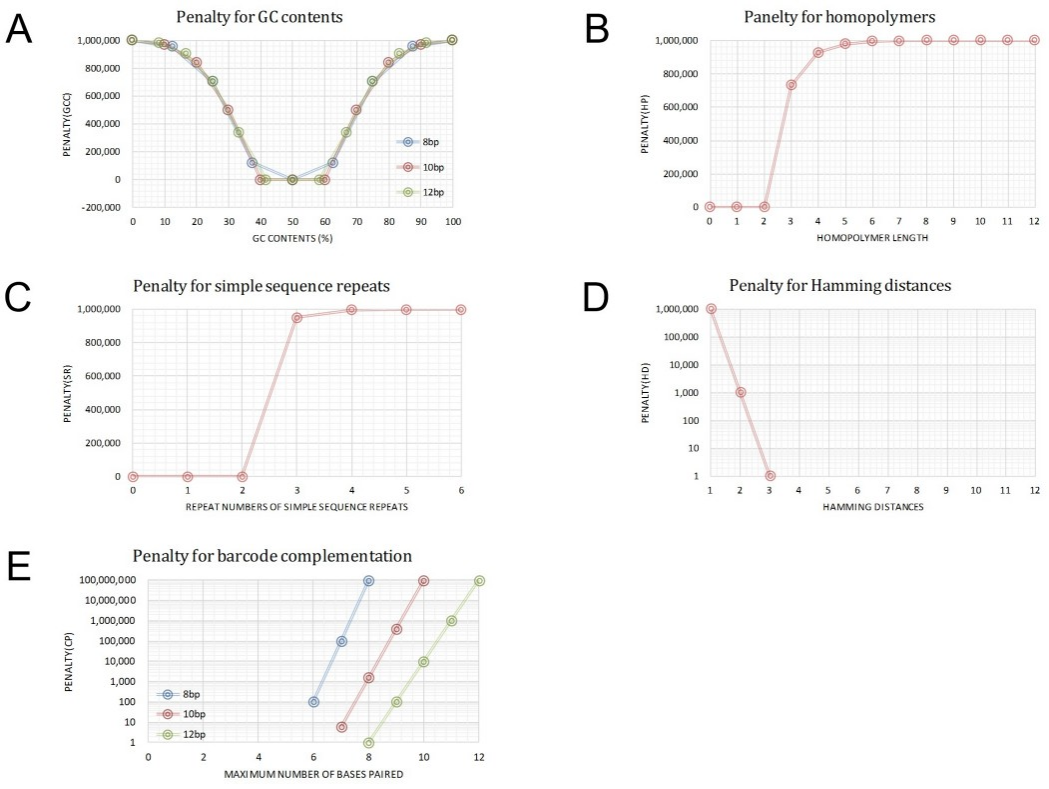
Distributions of penalty scores (P_GCC_, P_HP_, P_SR_, P_HD_, and P_CP_) for 5 barcode factors (GCC, HP, SR, HD, and CP). A, P_GCC_ distribution according to the percent GCC (*gcc*) at different *l* (8, 10, and 12). B, P_HP_ distribution according to the homopolymer length (*hp*) value. C, P_SR_ distribution according to the repeat number (*sr*) of simple sequence repeats. D, P_HD_ distribution according to the Hamming distances (*hd*). E, P_CP_ distribution according to the maximum number of bases paired (*cp*).

Fig 1A shows distributions of P_GCC_ according to the *gcc* range at different *l* (8, 10, and 12). Detailed values of *gcc*, *f*(*gcc*), $\bar{gcc}$, and P_GCC_ are represented in S1 Table.

### Penalty scores for HP (P_HP_) and SR (P_SR_)

We obtained two equations (Eqs (6) and (7)) by the curve fitting method from random distributions of *hp* and *sr*, respectively, along exponential curves, with modification as described earlier (S5b and S5c Fig, respectively).

$$f{\left(hp\right)}_{m}=461428\times {\text{e}}^{-1.31322\times hp}$$ (6)

$$f{\left(sr\right)}_{m}=15642980\times {\text{e}}^{-3.010114\times sr}$$ (7)

Since the number of barcodes with *hp* = 2 and *sr* = 2 were most frequently observed in distributions of *hp* (S4c Fig) and *sr* (S4d Fig), respectively, we set normalized *hp* score ($\bar{hp}$) at *hp* = 2 and normalized *sr* score ($\bar{sr}$) at *sr* = 2 as maximum values (10^6^). With a similar approach as that for $\bar{gcc}$ score, we defined equations to calculate $\bar{hp}$ and $\bar{sr}$ scores (Eqs (8) and (9), respectively).

$$\bar{hp}=\frac{f{\left(hp\right)}_{m}}{f{\left(hp=2\right)}_{m}}\times 10^{6}$$ (8)

$$\bar{sr}=\frac{f{\left(sr\right)}_{m}}{f{\left(sr=2\right)}_{m}}\times 10^{6}$$ (9)

Finally, P_HP_ and P_SR_ values were determined with Eqs (10) and (11), respectively, by subtracting $\bar{hp}$ and $\bar{sr}$ scores from 10^6^ with a modification to fix maximum penalty score to 10^6^ and to make the minimum penalty score a positive integer.

$${\text{P}}_{\text{HP}}=10^{6}-\bar{hp}+2$$ (10)

$${\text{P}}_{\text{SR}}=10^{6}-\bar{sr}+6$$ (11)

Fig 1B and 1C show distributions of P_HP_ and P_SR_, respectively. In detail, S2 Table represents the values of *f*(*hp*), $\bar{hp}$, and P_HP_ for HP (A); and *f*(*sr*), $\bar{sr}$, and P_SR_ for SR (B) according to increases in *hp* and *sr*, respectively.

### HD penalty score (P_HD_)

According to the assumption for P_HD_ score, we could represent its equation as shown in Eq (12), where the equation satisfying the conditions of *hd* = 1, 2, and 3 were obtained by curve fitting, as shown in S5d Fig.

$${\text{P}}_{\text{HD}}\{\begin{array}{c} 10^{9}\times {\text{e}}^{-6.907755\times hd}-7.524427\times 10^{-16},\;\;\text{if}\;hd=1,\;2,\;\text{or}\;3\\ 0,\;\;\text{if}\;hd\geq 4 \end{array}$$ (12)

As shown in Fig 1D and S3a Table, P_HD_ scores for barcodes in the range between *hd* = 1 and *hd* = 3 become 10^6^, 10^3^, and 1, respectively, and scores in the range of *hd*≥4 are zero (0).

### CP penalty score (P_CP_)

We introduced an increment of 10^2^ in the P_CP_ score system whenever *cp* increased by 1 to efficiently exclude barcodes that are close to or in a full complementary relationship. For barcodes with *l* = 12, P_CP_ scores at *cp* = 8, 9, 10, 11, and 12 become 1, 10^2^, 10^4^, 10^6^, and 10^8^, respectively. For *l*<8, we set P_CP_ scores to 0 as a minimum. Using this P_CP_ distribution, we determined the equation satisfying the conditions of $\tilde{cp}\geq 2/3\times l$ by using curve fitting approach (Eq (13) and S5e Fig), and treated P_CP_ score to 0 (zero) in the range of $\tilde{cp}\geq 2/3\times l$ according to the assumption.

$${\text{P}}_{\text{CP}}\{\begin{array}{c} 9.999999\times 10^{-17}\times {\text{e}}^{-4.60517\times \tilde{cp}}-0.0009216598,\;\;\;\text{if}\;\tilde{cp}\geq 2/3\times l\\ 0,\;\;\;\text{if}\;\tilde{cp}<2/3\times l \end{array}$$ (13)

In the equation, $\tilde{cp}$ was employed to obtain P_CP_ for different *l* (8 and 10) and was calculated using Eq (14). For barcodes with *l* = 12, $\tilde{cp}$ is equal to *cp*.

$$\tilde{cp}=\frac{cp}{l}\times 12$$ (14)

Fig 1E shows distributions of P_CP_ scores according to the *cp* at different *l*, which increase dramatically if *cp* is greater than the limit (2/3 of *l*) and become zero (0) if *cp* is less than the limit. Detailed values of the scores are also presented in S3b Table.

### Weights for penalty scores and α values for barcode exclusion

To determine final weights (w_1_, w_2_, w_3_, w_4_, and w_5_) for five barcode factors (GCC, HP, SR, HD, and CP), we examined P_DEC_ values for P_WT_ scores at total 4^5^ (= 1024) points on the five dimensional space from the origin (1, 1, 1, 1, 1) to the farthest point (20, 20, 20, 20, 20). We could observe the changes of penalty scores (P_GCC_, P_HP_, P_SR_, P_HD_, and P_CP_) for individual barcode factors together with P_WT_ between initial and best cycles at the median level as shown in S4 Table, which may be useful as a guideline to produce user own barcode sets by configuring one or more weights. We also obtained distributions of median P_DEC_ values according to the weights for the respective factors as shown in S6 Fig, where similar patterns of the distributions were observed between two intrinsic factors (GCC (a) and HP (b)), and between two pairwise factors (HD (d) and CP (e)). By comparing all P_DEC_ values for P_WT_ in S4 Table, we found the minimum value of 87.43% at the point of (20, 20, 20, 1, 1), revealing that higher intrinsic barcode factors (GCC, HP, and SR) and lower pairwise relationship factors (HD and CP) lead to higher P_DEC_ (i.e., minimized P_WT_). Therefore, we set the final weights to “20, 20, 20, 1, and 1,” respectively, as default values (Table 1).

**Table 1:** Initial and final weights determined in this study.

	Initial weights (A)	Final weights (B)	Folds (B/A)
w_1_	1	20	20.0
w_2_	1	20	20.0
w_3_	1	20	20.0
w_4_	1	1	1.0
w_5_	1	1	1.0

As shown in S5 Table, we could determine an appropriate α value of 0.2 by comparing P_WT_ values from the results of six different conditions, which gave us good results in many test runts. Therefore, the value was employed as a default in VFOS.

### Inputs and outputs of the VFOS program

Several input parameters are required to run this program: i) *l* (default: 12); ii) N (default: 10^5^); iii) α value (default: 0.2 [20%]); iv) penalty scores for five barcode factors, of which default values are set for barcodes with *l* = 12 (See S7 Fig for default penalty scores); and v) weights for penalty scores of GCC, HP, SR, HD, and CP (default: 20, 20, 20, 1, and 1, respectively). Default values are stored in parameter files (“param_general” for *l* and N; “param_alpha” for α value, “param_GCC, param_HP, param_SR, param_HD, and param_CP” for penalty scores for five barcode factors; and “param_weights” for weights). User-defined settings can be easily implemented by modifying the values of the parameters that are stored in their respective files. In addition, three files, “hd2_table, hd3_table, and hd4_table,” are mandatory for running the program as precalculated HD look-up-tables for short sequences with lengths of 2, 3, and 4, respectively. S7 Fig shows all of the input files containing respective default values.

Output files in text format are generated for each cycle, as well as at the initial state, and contain the selected barcodes and information on barcode length, the number of barcodes generated, and total penalty scores for the five barcode factors, as shown in S8 Fig.

### Performance of the VFOS program

To test the performance of VFOS, we set up four conditions with different weights, in which we were able to obtain optimal barcode sets from the conditions. Users should be aware that the term “optimal” means finally optimized under a certain condition defined by parameters. Therefore, if different penalty scores and weights are given, totally different barcodes will be produced based on the parameters.

#### Results from the default setting

**Fig 2:**
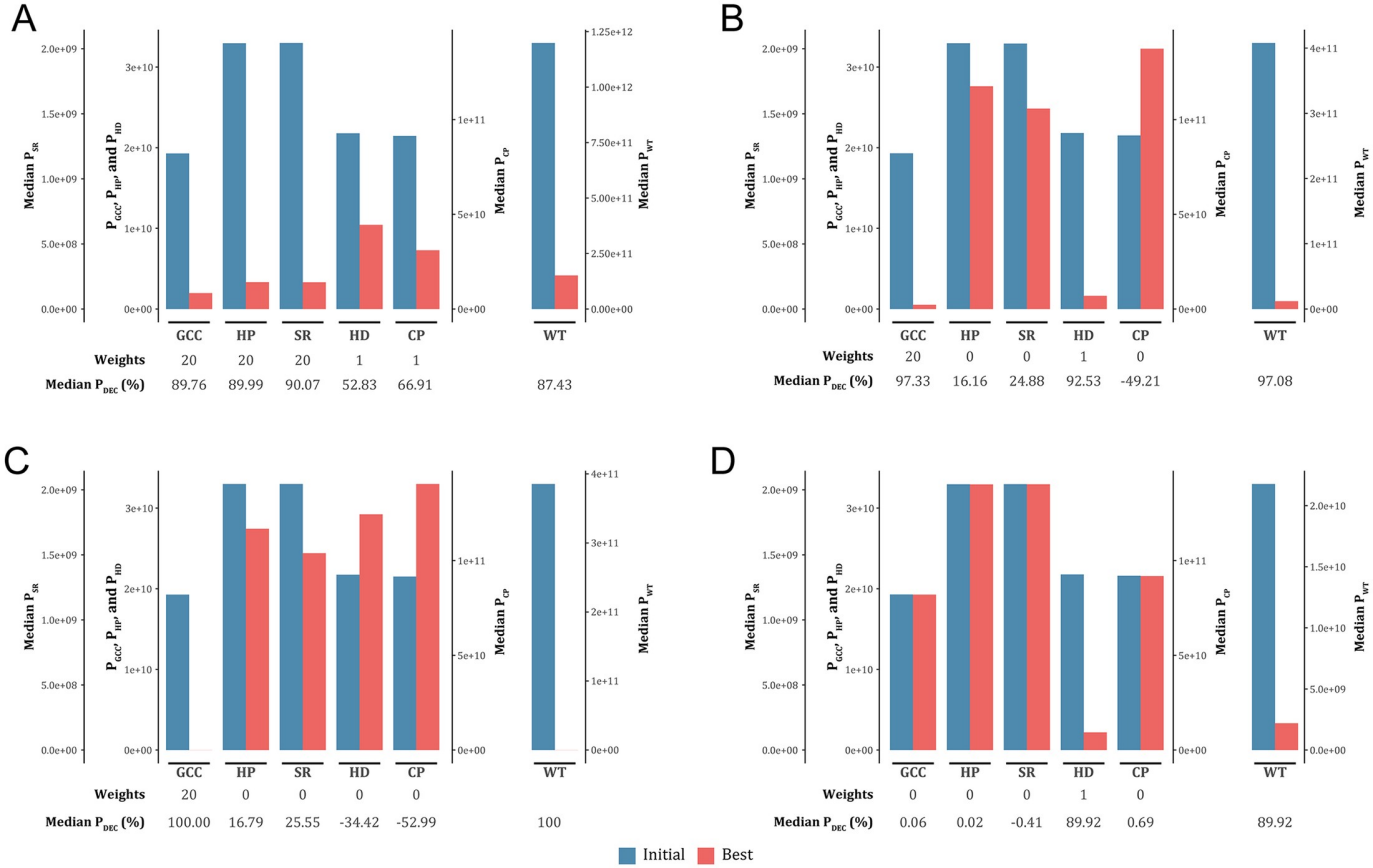
Results from test runs in four different conditions. All five barcode factors (A), two factors (GCC and HD; B), one factor (GCC; C) and another single factor (HD; D) were considered in the conditions for selecting an optimal barcode set in each condition. Steel blue and tomato red bars represent penalty scores of the barcode factors in initial and final sets, respectively. P_GCC_, P_HP_, P_SR_, P_HP_, and P_CP_ represent penalty scores for GCC, HP, SR, HD, and CP, respectively. P_DEC_ means a percent decrease in penalty scores obtained by comparing results between initial and best cycles.

Since the default setting showed minimized penalty scores for all barcode factors, the setting can be used for general use to obtain an optimal barcode set. As shown in Fig 2A, S6a Table, and S9a Fig, our test results in this setting revealed that initial P_WT_ values (P_WTa_) from all 1000 runs ranged from 1.189E+12 to 1.213E+12 (median P_WTa_ = 1.198E+12), and the lowest P_WT_ values (P_WTb_) were observed between 1.466E+11 and 1.586E+11 (median P_WTb_ = 1.506E+11). Consequently, P_DEC_ values of P_WT_ scores appeared between 86.73% and 87.82% (median P_DEC_ = 87.43%), revealing an overall performance of VFOS in this setting of 87.43%. When we examined P_DEC_ values of penalty scores for individual barcode factors (P_GCCt_, P_HPt_, P_SRt_, P_HDt_, and P_CPt_), their median values were 89.76% for GCC, 89.99% for HP, 90.07% for SR, 52.83% for HD, and 66.91% for CP. The P_DEC_ values of GCC, HP, and SR were greater than the overall performance, and those for HD and CP were smaller than it. From the results, we confirmed that many barcodes in the final set were selected to have more balanced GCCs, shorter HPs, shorter SRs, higher HDs, and lower CPs toward the direction intended in previous studies [18–23] and primer design [25].

#### Results from the first user-defined setting

In the first user-defined setting (Fig 2B, S6b Table, and S9b Fig), we selected two barcode factors of GCC and HD as monitoring targets to obtain a barcode set with minimized P_GCCt_ and P_HDt_. For the purpose, we adjusted the weights of their penalty scores as follows: w_1_ = 20.0; w_2_ = 0; w_3_ = 0; w_4_ = 1.0; and w_5_ = 0. Accordingly, P_WT_ score was calculated as a summed value of P_GCCt_ and P_HDt_. In this setting, P_WTa_ was observed between 4.047E+11 and 4.112E+11 (median P_WTa_ = 4.078E+11), and P_WTb_ ranged from 1.115E+10 to 1.248E+10 (median P_WTb_ = 1.192E+10). P_DEC_ values of P_WT_ scores appeared between 96.94% and 97.27% (median P_DEC_ = 97.08%), revealing an overall performance of VFOS in this setting of 97.08%. Because only two barcode factors were considered in this condition, the performance of this condition was highly improved, compared to the default setting. Furthermore, penalty scores for GCC and HD were greatly decreased to 97.33% and 92.53% at the median level. In contrast, penalty scores for other barcode factors (HP, SR, and CP) were either slightly decreased or increased (16.16%, 24.88%, and -49.21%, respectively, at the median level).

#### Results from the second user-defined setting

In the second user-defined setting (Fig 2C, S6c Table, and S9c Fig), a single barcode factor of GCC was selected as a monitoring target, and a weight (w_1_) was given as a positive value (20.0). In this condition, P_WTb_ scores (= P_GCCb_) decreased to 0 (P_DEC_ of 100%), meaning that *gcc* values of all barcodes were in the range between 40% and 60%. In addition, penalty scores for other barcode factors (HP, SR, HD, and CP) slightly decreased or greatly increased (16.79%, 25.55%, -34.42% and -52.99%, respectively, at the median level). From these results, we could note a positive relationship between GCC and HP/SR and a negative one between GCC and HD/CP.

#### Results from the third user-defined setting

In the third user-defined setting (Fig 2D, S6d Table, and S9d Fig), the barcode factor HD was selected as a monitoring target, and a weight (w_4_) was given as a positive value (1.0). P_WT_ scores (= P_HDt_) dropped to 2.177E+10 at the median level (median P_DEC_ of 89.92%). Unlike the case of GCC, we discovered that HD showed no or little association with other factors.

#### Total cycles and running time per cycle

We examined the running time of VFOS for selecting 10^5^ optimal barcode sets in two different computing environments: i) a dual-core desktop PC and ii) a node cluster system capable of 20 threads. A similar number of cycles per run were recorded in the range between eight and 43 for both systems. The calculated running times per cycle were ~ 722 sec (~13 min) in the former system and ~118 sec (~2 min) in the latter system. For a run of 50 cycles, the expected run time would be ~10 hours 50 min and ~ 1 hour 40 min for the two systems, respectively.

### Possible examples of misclustering of barcodes

We found two possible examples of inaccurate interpretation in the data analysis procedure that true variants could occasionally be missed out due to misclustering of barcodes when random barcodes were used to detect low frequent variants, which were related to HD between two barcodes and HPs in barcodes, respectively.

The first example was found in the analysis of a targeted DNA sequencing data set from lung cancer liquid biopsy samples with a L858R mutation of T>G transversion in the epidermal growth factor receptor gene, in which random barcodes were used for accurate identification of mutations (unpublished data). In a sample, the depth-based frequency of the mutation was detected at 0.51% (15 of 2922; forward 7 and reverse 8 barcodes; S9a Fig). However, its barcode-based frequency was identified at 0.75% (13 of 1739; forward 6 and reverse 7 barcodes; S9b Fig). In addition, two barcodes with the relationship *hd* = 1 (“GGGGCAGTCGGG” vs. “GGGGCAGACGGG”) were observed in both forward and reverse directions of the reads with the mutation. The corresponding reads were not clustered into the same group due to differences in their length and sequence. However, if the reads were clustered into the same group due to an unexpected mutation in one of their barcodes, its barcode-based frequency would be lowered to 0.63% (11 of 1737; forward 5 and reverse 6 barcodes; S9c Fig).

The second example was found in previous research [6], in which barcodes with *l* = 12 were used for variant identification from liquid biopsy samples of cancer patients by sequencing DNA fragments using the Ion Torrent platform. The sequencing system is known to have high insertion and deletion error rates, especially in HP regions [29, 30]. If the possible errors are not considered in barcode clustering, misclustering of barcodes will occur. For this reason, Kukita et al. [6] employed an approach in which barcodes with *l* = 11 and 13 that only differed from a barcode with *l* = 12 by the insertion or deletion of a single base were grouped with the corresponding barcode of *l* = 12, as shown in S7 Table.

### Comparison with other tools

There are four features of VFOS distinct from two other tools (DNABarcodes [22] and FreeBarcodes [23]). The first feature of VFOS is flexibility in program running that can accept user-own parameters of the number of barcodes created, weights, α value, and even penalty scores. Especially, different combinations of weights could be applied to generate different barcode sets as shown in Fig 2 and S4 Table. The second feature is that VFOS can create a large amount of barcodes suitable for molecular barcoding. When we created barcodes with *l* = 12 using DNABarcodes and FreeBarcodes, 2857 and 98536 barcodes were generated from DNABarcodes with MD (dist) of 3 and 5, respectively, and 178 and 17213 barcodes with *l* = 12 were obtained from FreeBarcodes with number of errors (num_errors) of 1 and 2, respectively. Unlike the programs, we could generate more than 100000 barcodes using VFOS.

In addition, when we generated the same number of barcodes (178, 2857, 17213, and 98536) using VFOS and then compared barcode sets from VFOS and the other tools with 6 criteria (GCC, HP, SR, HD, LD, and CP) as shown in S8–S13 Tables, we could observe that VFOS produced barcodes with balanced *gcc* (S11a Fig and S8 Table), shorter *hp* (S11b Fig and S9 Table), and higher *hd* (S11c Fig and S10 Table) and *ld* (S11d Fig and S11 Table). The results revealed that VFOS has comparable performance to the two tools, although inclusion of a small portion of non-allowed or unfavorable barcodes were permitted for generating large amounts of barcodes in limited barcode lengths (*l*). In comparison with CP distributions, VFOS also produced barcodes with more concentrated *cp* toward the peak (*cp* = 5), which was affected to reduce barcodes with unfavorable *cp*, especially at *cp* = 12 that means perfect reverse-complementary between barcodes (S12 Table).

**Fig 3:**
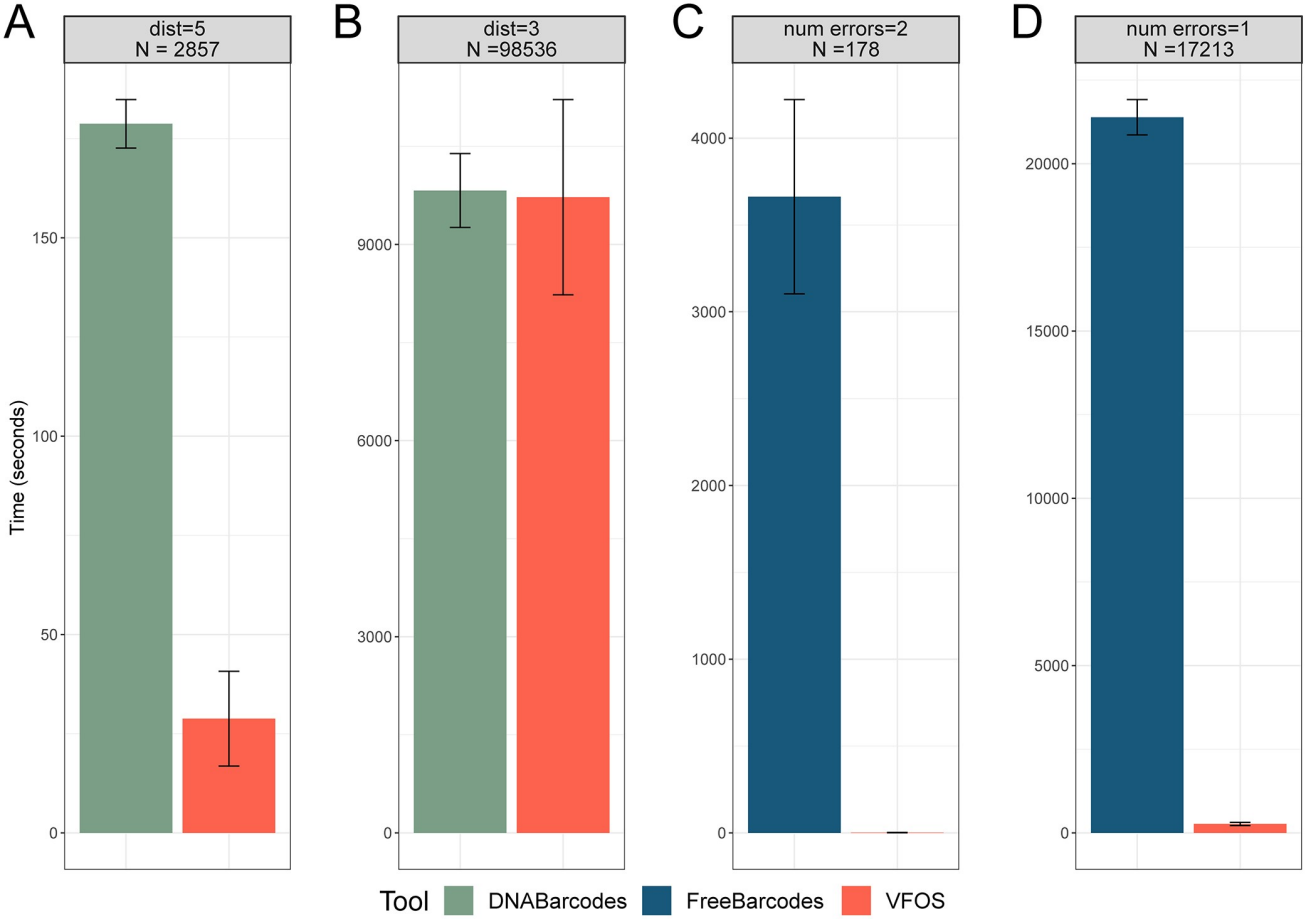
Comparison of computation time for barcode generation between VFOS and other two tools (DNABarcodes and FreeBarcodes). Computation time between VFOS and DNABarcodes with 2857 (A) and 98536 barcode generation (B), and between VFOS and FreeBarcodes with 178 (C) and 17213 barcode generation (D).

The third feature is that only VFOS considers SRs that are often observed in barcode sequence as a typical repeat consisting of dinucleotides. When SR distributions were compared from the results, VFOS showed that many barcodes have shorter *sr* than those from the two tools (S11e Fig and S13 Table), thereby enabling to reduce possible error sources in PCR amplification and sequencing steps. The fourth feature is that VFOS showed faster or similar performance over computation time compared to the other tools as shown in Fig 3. In comparison with DNABarcodes, VFOS was faster or similar computation time when we produced 2857 (3A) and 98536 barcodes (3B). In comparison with FreeBarcodes, VFOS showed overwhelmingly faster performance when we created 178 (3A) and 17213 barcodes (3D).

## Discussions

We developed VFOS to provide users with a program that can generate an optimal set of barcode sequences for NGS with unique molecular identifier (UMI) (a.k.a. molecular barcoding) techniques. As the purpose of UMI sequencing is to tag every single DNA fragment in the library preparation step, the number of barcodes should be large enough (e.g., >1 million) in most cases. We would like to note that previous algorithms or tools [18–23] also aimed to design optimal sets of DNA barcodes, which are more applicable for multiplex sequencing. In multiplex sequencing, barcodes are used to discriminate samples that are being sequenced in a same NGS lane, the number of which is a few hundred at most. We found that none of the tools was applicable to UMI due to the limitation in the output size (<100000) for typical barcode lengths (8 to 12). Furthermore, we showed that VFOS has comparable performance by comparing with DNABarcodes [22] and FreeBarcodes [23] in the same condition (*l* = 12), although different design was applied in VFOS. In addition, VFOS has flexibility in program running, the consideration of SRs as a barcode factor, and fast computation time in comparison with the tools.

In the implementation, we applied many computational techniques and heuristics to increase the speed of VFOS, *i*.*e*., pre-calculation of frequently used values and the use of look-up-tables, which enabled the generation of large barcode sets of multi-million scales. Multi-threading was also implemented and effective in speeding up the program (see Results). In addition, we noted that many users do not need to generate new barcode sets again, but can reuse any of those that are already optimized. For such users, we provide several sets of prebuilt barcodes with different conditions (e.g., barcode length and parameters) in the program webpage [34]. These sets can be downloaded and instantly applied for sequencing, reducing time and cost for users substantially.

With the rapid development of sequencing technologies, molecular barcoding is now widely in various systems. Sometimes, complex designs are required for barcoding. For example, the Chromium platform (10x Genomics Inc.) exploits a double barcoding system that consists of the 10x-barcodes and UMI, which identify the single cell and molecule, respectively [35]. In most cases, sample index is further attached to the libraries, presenting three different barcode systems in a read. We expect that further optimization can be done for multiple barcoding, such as considering barcodes of longer lengths and interactions among different barcoding systems.

## Conclusions

We have developed a versatile and fast tool (VFOS) for selecting oligonucleotide subsets that can be utilized in the detection of low-frequent somatic mutations, in the quantification of gene- or transcript-level expression, and in single-cell sequencing. Although our *in silico* work requires experimental validation, we demonstrated the versatility and fast performance of VFOS by providing optimal barcode sets for various conditions. We also showed that VFOS has comparable performance, flexibility in program running, consideration of SRs, and fast computation time in comparison with other two tools (DNABarcodes and FreeBarcodes). Therefore, we expect that many researchers will opt to apply the program for selecting optimal barcode sets during their experiments, including next-generation sequencing.

## Availability and requirements

Project name: VFOS

Project home page: https://sourceforge.net/projects/vfos/

Operating system: Linux

Programming language: C++

Other requirements: None

License: Non-Commercial Research Use Only

## Supporting information

S1 Fig Calculation of maximum number of base paired (*cp*) between two barcodes. (PPTX)

S2 Fig Distributions of five barcode factors (GCC, HP, SR, HD, and CP) observed in random barcodes with *l* = 8. (PPTX)

S3 Fig Distributions of five barcode factors (GCC, HP, SR, HD, and CP) observed in random barcodes with *l* = 10. (PPTX)

S4 Fig Distributions of five barcode factors (GCC, HP, SR, HD, and CP) observed in random barcodes with *l* = 12. (PPTX)

S5 Fig Curve fitting results that used to derive penalty scores for five barcode factors. (PPTX)

S6 Fig Distributions of P_DEC_ values according to the weights (w_1_, w_2_, w_3_, w_4_, and w_5_). (PPTX)

S7 Fig Parameters setting that produces 10000 barcodes with *l* = 12 using default penalty scores and weights. (PPTX)

S8 Fig Example results in an output file in text format. The results comprise barcode length, number of barcodes, total penalty scores of the barcode set, and selected barcodes ordered alphabetically.
(PPTX)

S9 Fig Histograms of penalty scores (P_GCC_, P_HP_, P_SR_, P_HD_, P_CP_, and P_WT_) at initial and best cycles for Fig 2. (PPTX)

S10 Fig Schematic representation of mutation frequencies of EGFR L858R. (PPTX)

S11 Fig Comparison results between VFOS and other programs (DNABarcodes and FreeBarcodes). (PPTX)

S1 Table Penalty scores for GCC (P_GCC_) depending on the lengths of barcodes (8, 10, and 12 nt). (XLSX)

S2 Table Penalty scores for HP (P_HP_) and SR (P_SR_) according to the lengths of HP (*hp*) and the repeat numbers of SR (*sr*) regardless of the lengths of barcodes. P_HP_ for *hp* = 1 and P_SR_ for *sr* = 1 are the same with the cases of *hp* = 2 and *sr* = 2, respectively.
(XLSX)

S3 Table Penalty scores for two pairwise barcode factors (P_HD_ and P_CP_) according to barcode length. (XLSX)

S4 Table Penalty scores (P_GCC_, P_HP_, P_SR_, P_HD_, P_CP_, and P_WT_) and their P_DEC_ values according to the weights. (XLSX)

S5 Table Decreasing patterns of P_WT_ scores in 6 conditions with individual values (0.05, 0.1, 0.2, 0.3, 0.4, and 0.5). The lowest P_WT_ score appeared at 5th cycle when α was set to 0.2.
(XLSX)

S6 Table Comparison with performance results between default and user-define modes. In user-defined mode, it was tested to a barcode set having minimized penalty scores for GCC and HD (P_GCC_ and P_HD_).
(XLSX)

S7 Table Clustering examples of barcodes considered that insertion or deletion of a single base occurred. Different bases were underlined in the barcode sequence. Note that this example was adopted from Kukita et al.
(XLSX)

S8 Table GC content (GCC) distribution for barcodes with *l* = 12. (XLSX)

S9 Table Homopolymer (HP) distribution for barcodes with *l* = 12. (XLSX)

S10 Table Hamming distance (HD) distribution for barcodes with *l* = 12. (XLSX)

S11 Table Levenshtein distance (LD) distribution for barcodes with *l* = 12. (XLSX)

S12 Table Complementarity (CP) distribution for barcodes with *l* = 12. (XLSX)

S13 Table Simple sequence repeat (SR) distribution for barcodes with *l* = 12. (XLSX)

## Abbreviations

α value: Percentage of barcodes excluded from a barcode set
$\tilde{cp}$: Converted value of *cp* according to different *l*
CP: Complementarity
cp: Maximum number of bases paired
$\bar{gcc}$: Normalized *gcc* score
GCC: GC contents
*gcc*: Percentage of GCC
$\bar{hp}$: Normalized *hp* score
HD: Hamming distance
*hd*: HD-based edit distance
HP: Homopolymer
hp: Length of HPs
*l*: Barcode length
*ld*: LD-based edit distance
LD: Levenshtein distance
MD: Minimum distance
N: Number of barcodes to be generated
P_a_: One of six total penalty scores (P_GCCt_, P_HPt_, P_SRt_, P_HDt_, P_CPt_ or P_WT_) during the initial cycle (= 0^th^ cycle)
P_b_: One of six total penalty scores (P_GCCt_, P_HPt_, P_SRt_, P_HDt_, P_CPt_ or P_WT_) during the best cycle (a cycle showing the lowest P_WT_ value in a run)
P_CP_: Penalty score for CP of a barcode
P_CP*i*_: Penalty score for CP of the *i*-th barcode
P_CPt_: Total penalty score for CP of a barcode set
P_DEC_: Percent decrease between P_a_ and P_b_
P_EX_: Probability of barcode exclusion
P_GCC_: Penalty score for GCC of a barcode
P_GCC*i*_: Penalty score for GCC of the *i*-th barcode
P_GCCt_: Total penalty score for GCC of a barcode set
P_HD_: Penalty score for HD of a barcode
P_HD*i*_: Penalty score for HD of the *i*-th barcode
P_HDt_: Total penalty score for HD of a barcode set
P_HP_: Penalty score for HPs of a barcode
P_HP*i*_: Penalty score for HP of the *i*-th barcode
P_HPt_: Total penalty score for HP of a barcode set
P_SR_: Penalty score for SRs of a barcode
P_SR*i*_: Penalty score for SR of the *i*-th barcode
P_SRt_: Total penalty score for SR of a barcode set
P_T*i*_: Weighted total penalty scores for GCC, HP, SR, HD, and CP of the *i*-th barcode
P_WT_: Weighted total penalty score of a barcode set
P_WTa_: P_WT_ value during the initial cycle (= 0^th^ cycle)
P_WTb_: P_WT_ value during the best cycle (a cycle showing the lowest P_WT_ value in a run)
$\bar{sr}$: Normalized *sr* score
*sr*: Repeat number of SRs
SR: Simple sequence repeat with repeated units of dinucleotides
w1: Weight for P_GCC_
w2: Weight for P_HP_
w3: Weight for P_SR_
w4: Weight for P_HD_
w5: Weight for P_CP_
