# Supplementary material for: Development of a program for in silico optimized selection of oligonucleotide-based molecular barcodes
Source: PLoS One. 2021 Feb 18;16(2):e0246354. doi: 10.1371/journal.pone.0246354 (PMC7891705; doi:10.1371/journal.pone.0246354)
Supplement: S1 Fig — (PPTX) [file pone.0246354.s001.pptx]

## Slide 1
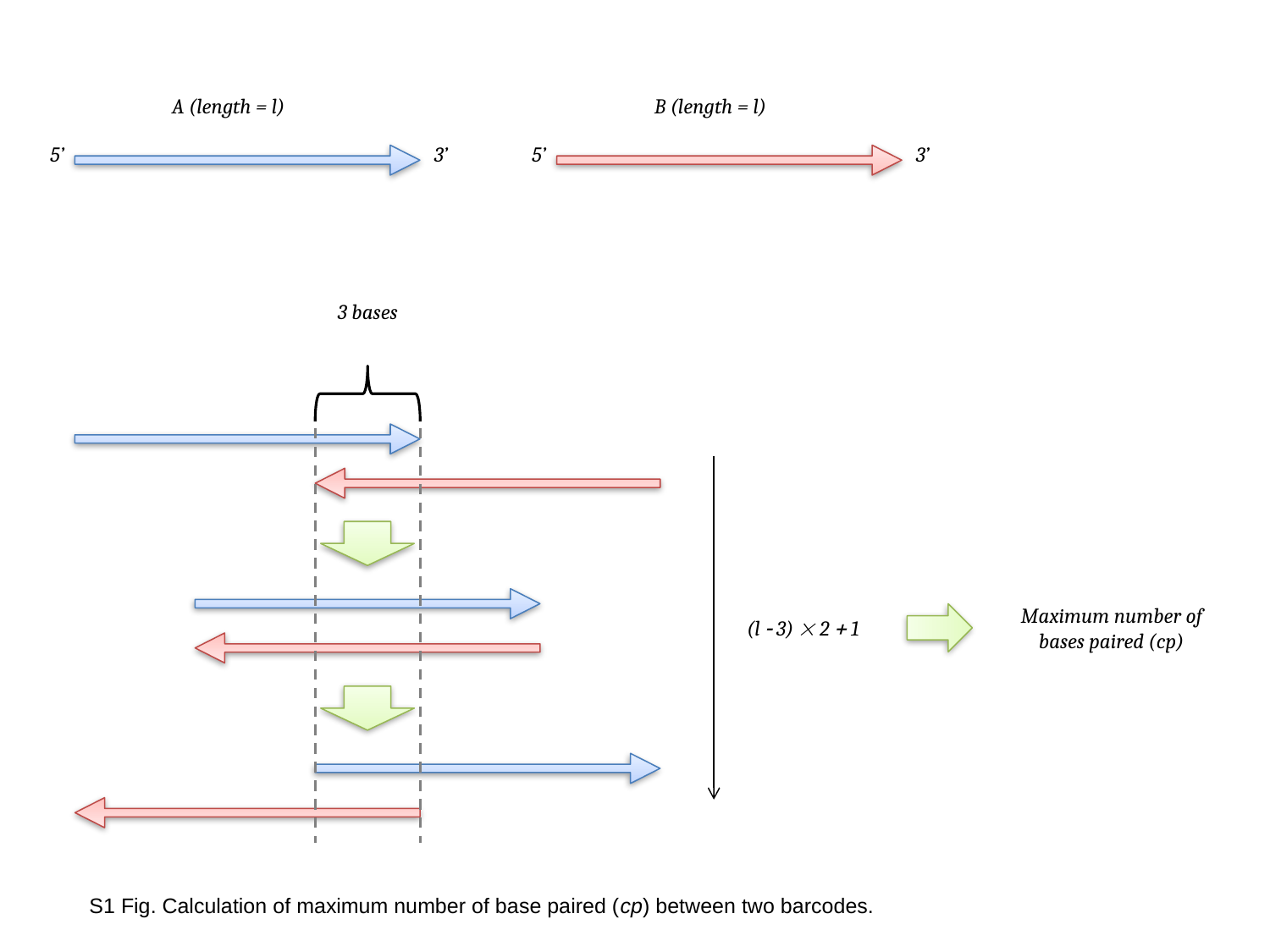

A (length = l)
5’
3’
B (length = l)
5’
3’
3 bases
Maximum number of bases paired (cp)
(l − 3) × 2 + 1
S1 Fig. Calculation of maximum number of base paired (cp) between two barcodes.
