# Supplementary material for: Development of a program for in silico optimized selection of oligonucleotide-based molecular barcodes
Source: PLoS One. 2021 Feb 18;16(2):e0246354. doi: 10.1371/journal.pone.0246354 (PMC7891705; doi:10.1371/journal.pone.0246354)
Supplement: S4 Fig — (PPTX) [file pone.0246354.s004.pptx]

## Slide 1
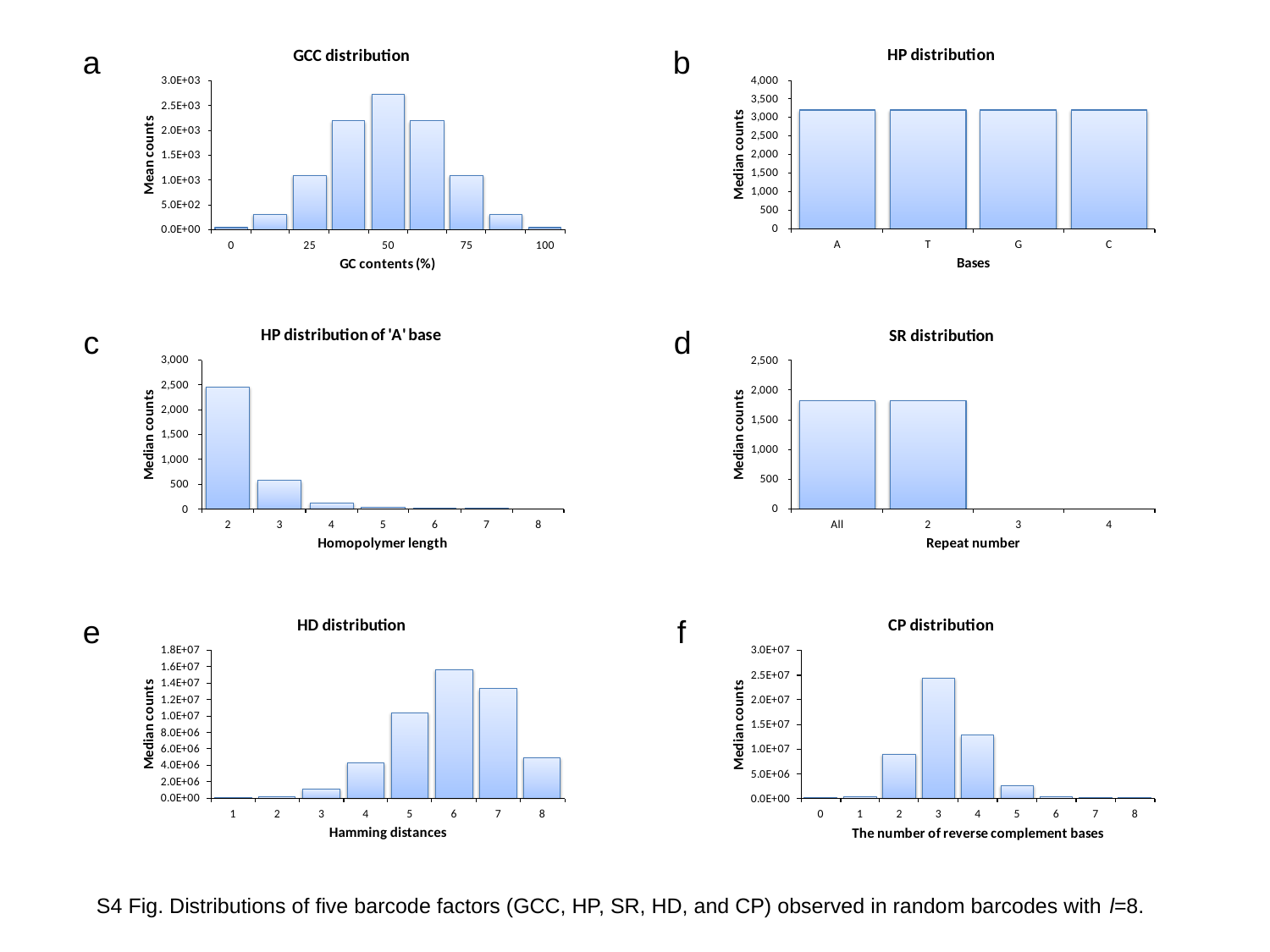

a
b
c
d
e
f
S4 Fig. Distributions of five barcode factors (GCC, HP, SR, HD, and CP) observed in random barcodes with l=8.
