# Supplementary material for: Development of a program for in silico optimized selection of oligonucleotide-based molecular barcodes
Source: PLoS One. 2021 Feb 18;16(2):e0246354. doi: 10.1371/journal.pone.0246354 (PMC7891705; doi:10.1371/journal.pone.0246354)
Supplement: S5 Fig — (PPTX) [file pone.0246354.s005.pptx]

## Slide 1
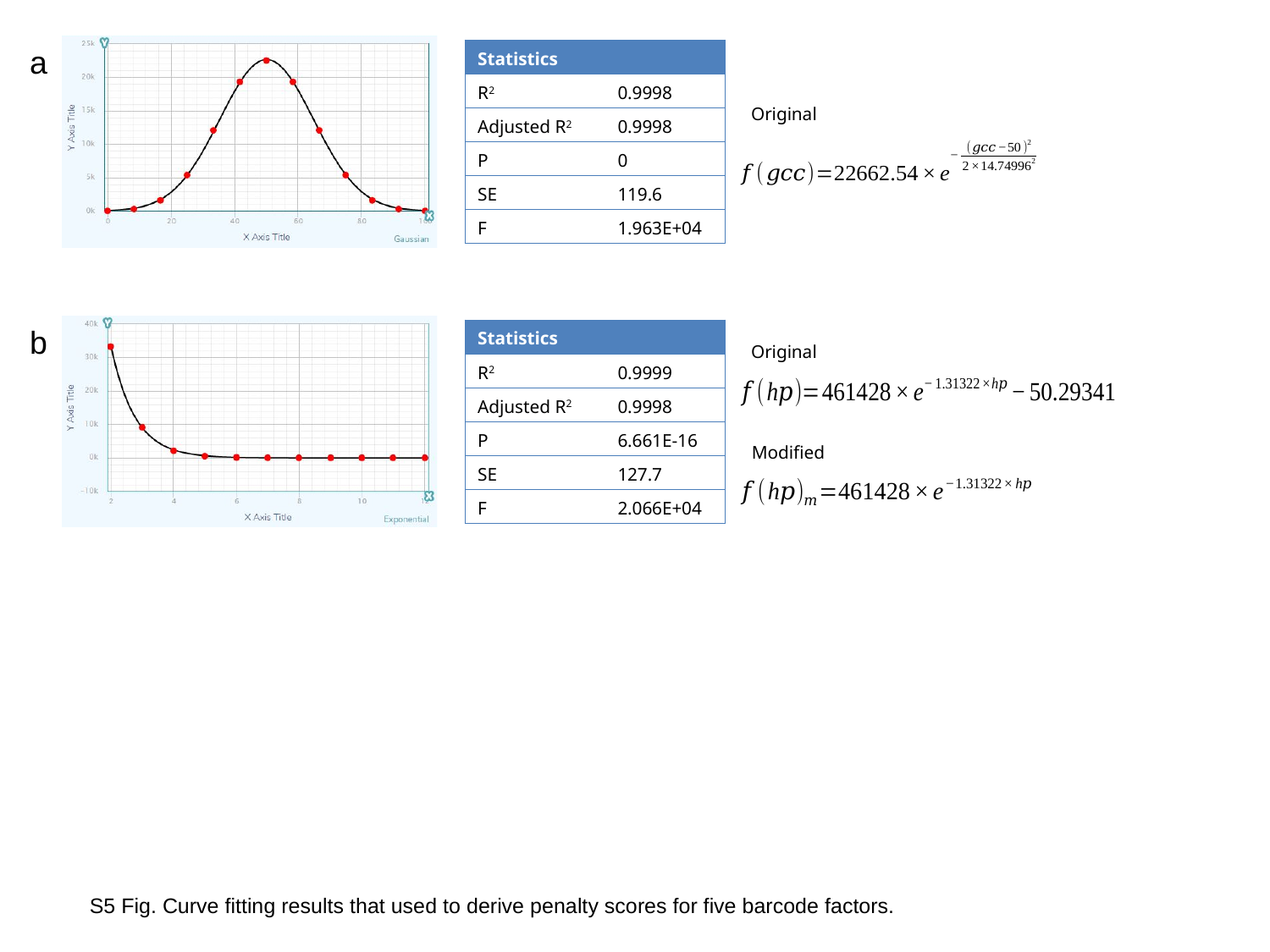

a
| Statistics | |
| --- | --- |
| R2 | 0.9998 |
| Adjusted R2 | 0.9998 |
| P | 0 |
| SE | 119.6 |
| F | 1.963E+04 |
Original
b
| Statistics | |
| --- | --- |
| R2 | 0.9999 |
| Adjusted R2 | 0.9998 |
| P | 6.661E-16 |
| SE | 127.7 |
| F | 2.066E+04 |
Original
Modified
S5 Fig. Curve fitting results that used to derive penalty scores for five barcode factors.

## Slide 2
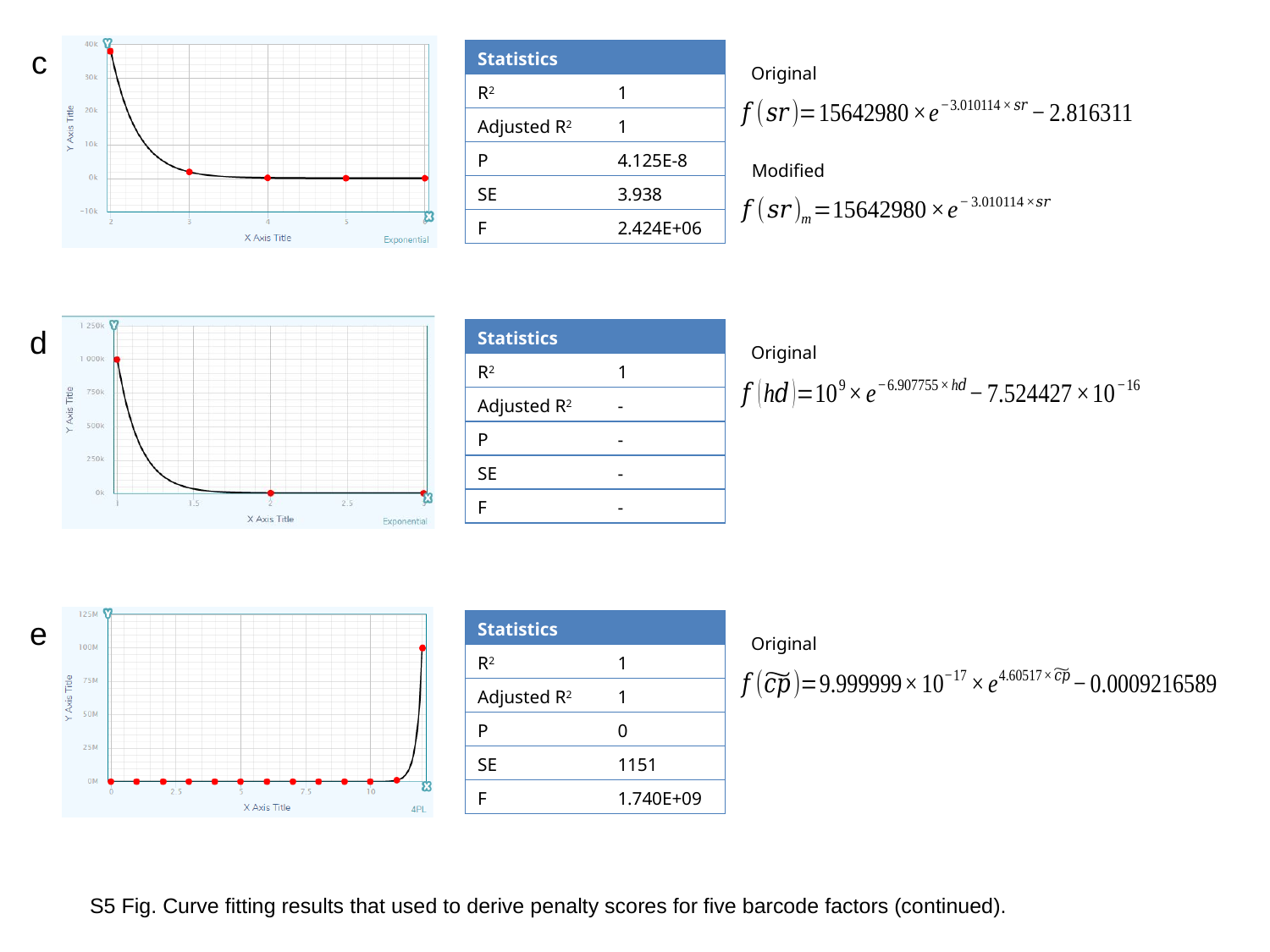

c
| Statistics | |
| --- | --- |
| R2 | 1 |
| Adjusted R2 | 1 |
| P | 4.125E-8 |
| SE | 3.938 |
| F | 2.424E+06 |
Original
Modified
d
| Statistics | |
| --- | --- |
| R2 | 1 |
| Adjusted R2 | - |
| P | - |
| SE | - |
| F | - |
Original
e
| Statistics | |
| --- | --- |
| R2 | 1 |
| Adjusted R2 | 1 |
| P | 0 |
| SE | 1151 |
| F | 1.740E+09 |
Original
S5 Fig. Curve fitting results that used to derive penalty scores for five barcode factors (continued).
