# Supplementary figures and images for: Development of a program for in silico optimized selection of oligonucleotide-based molecular barcodes
Source: PLoS One. 2021 Feb 18;16(2):e0246354. doi: 10.1371/journal.pone.0246354 (PMC7891705; doi:10.1371/journal.pone.0246354)

## Slide 1
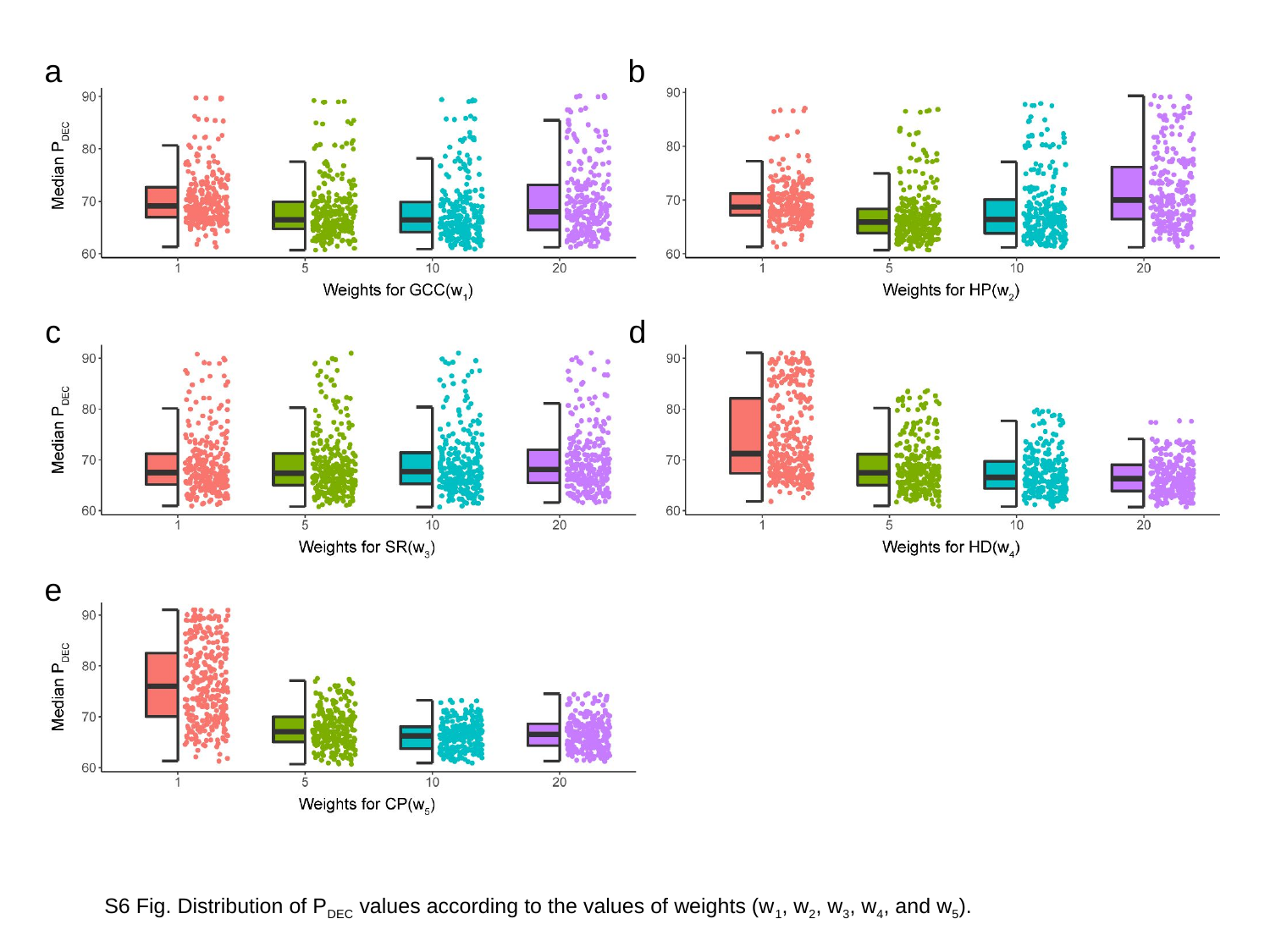

a
b
c
d
e
S6 Fig. Distribution of PDEC values according to the values of weights (w1, w2, w3, w4, and w5).

Supplement: S6 Fig — (PPTX) [file pone.0246354.s006.pptx]
