# Supplementary material for: Development of a program for in silico optimized selection of oligonucleotide-based molecular barcodes
Source: PLoS One. 2021 Feb 18;16(2):e0246354. doi: 10.1371/journal.pone.0246354 (PMC7891705; doi:10.1371/journal.pone.0246354)
Supplement: S7 Fig — (PPTX) [file pone.0246354.s007.pptx]

## Slide 1
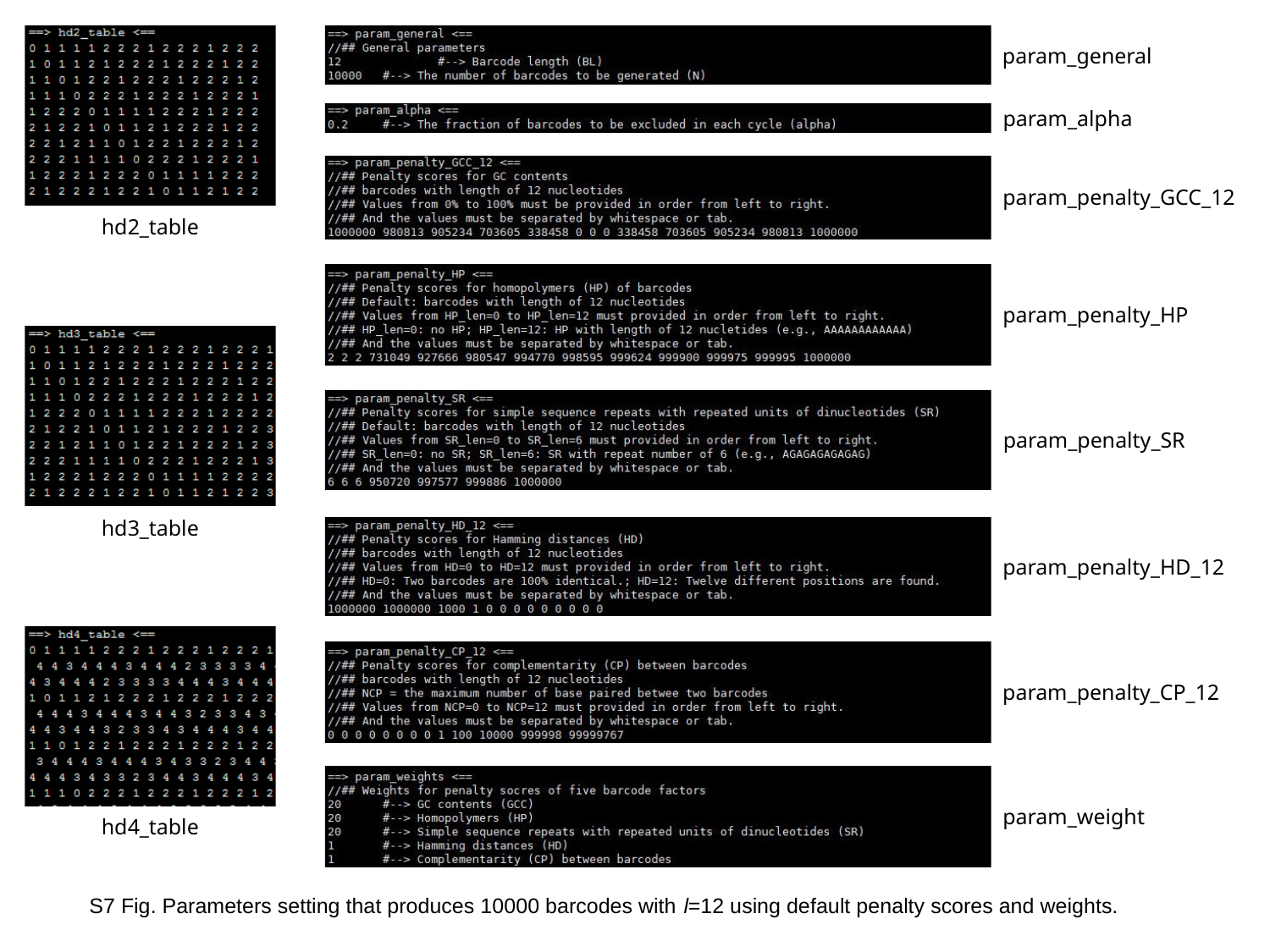

param_general
param_alpha
param_penalty_GCC_12
hd2_table
param_penalty_HP
param_penalty_SR
hd3_table
param_penalty_HD_12
param_penalty_CP_12
param_weight
hd4_table
S7 Fig. Parameters setting that produces 10000 barcodes with l=12 using default penalty scores and weights.
