# Supplementary material for: Development of a program for in silico optimized selection of oligonucleotide-based molecular barcodes
Source: PLoS One. 2021 Feb 18;16(2):e0246354. doi: 10.1371/journal.pone.0246354 (PMC7891705; doi:10.1371/journal.pone.0246354)
Supplement: S8 Fig — The results comprise barcode length, number of barcodes, total penalty scores of the barcode set, and selected barcodes ordered alphabetically. (PPTX) [file pone.0246354.s008.pptx]

## Slide 1
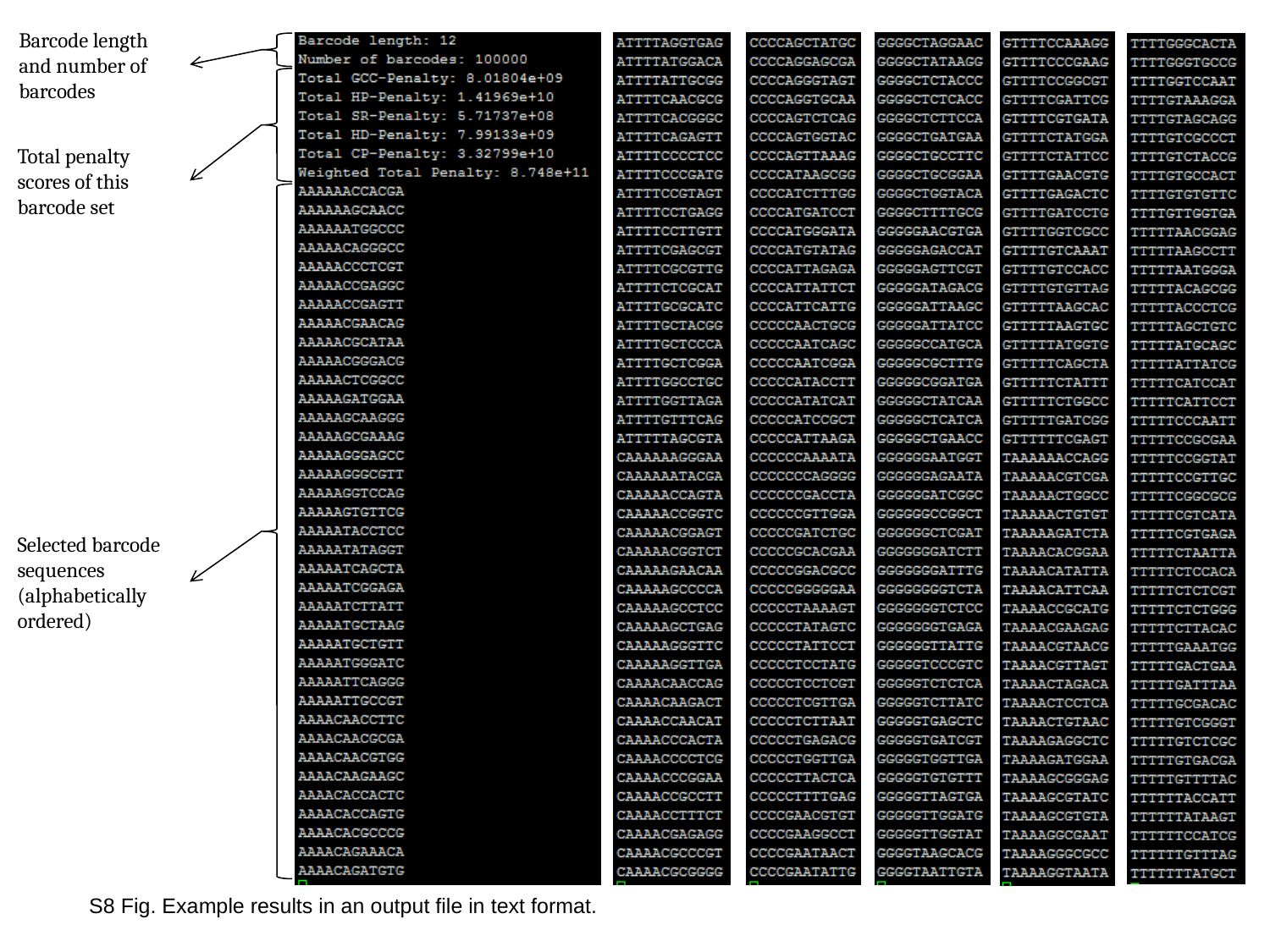

Barcode length and number of barcodes
Total penalty scores of this barcode set
Selected barcode sequences (alphabetically ordered)
S8 Fig. Example results in an output file in text format.
