# Supplementary material for: Development of a program for in silico optimized selection of oligonucleotide-based molecular barcodes
Source: PLoS One. 2021 Feb 18;16(2):e0246354. doi: 10.1371/journal.pone.0246354 (PMC7891705; doi:10.1371/journal.pone.0246354)
Supplement: S9 Fig — (PPTX) [file pone.0246354.s009.pptx]

## Slide 1
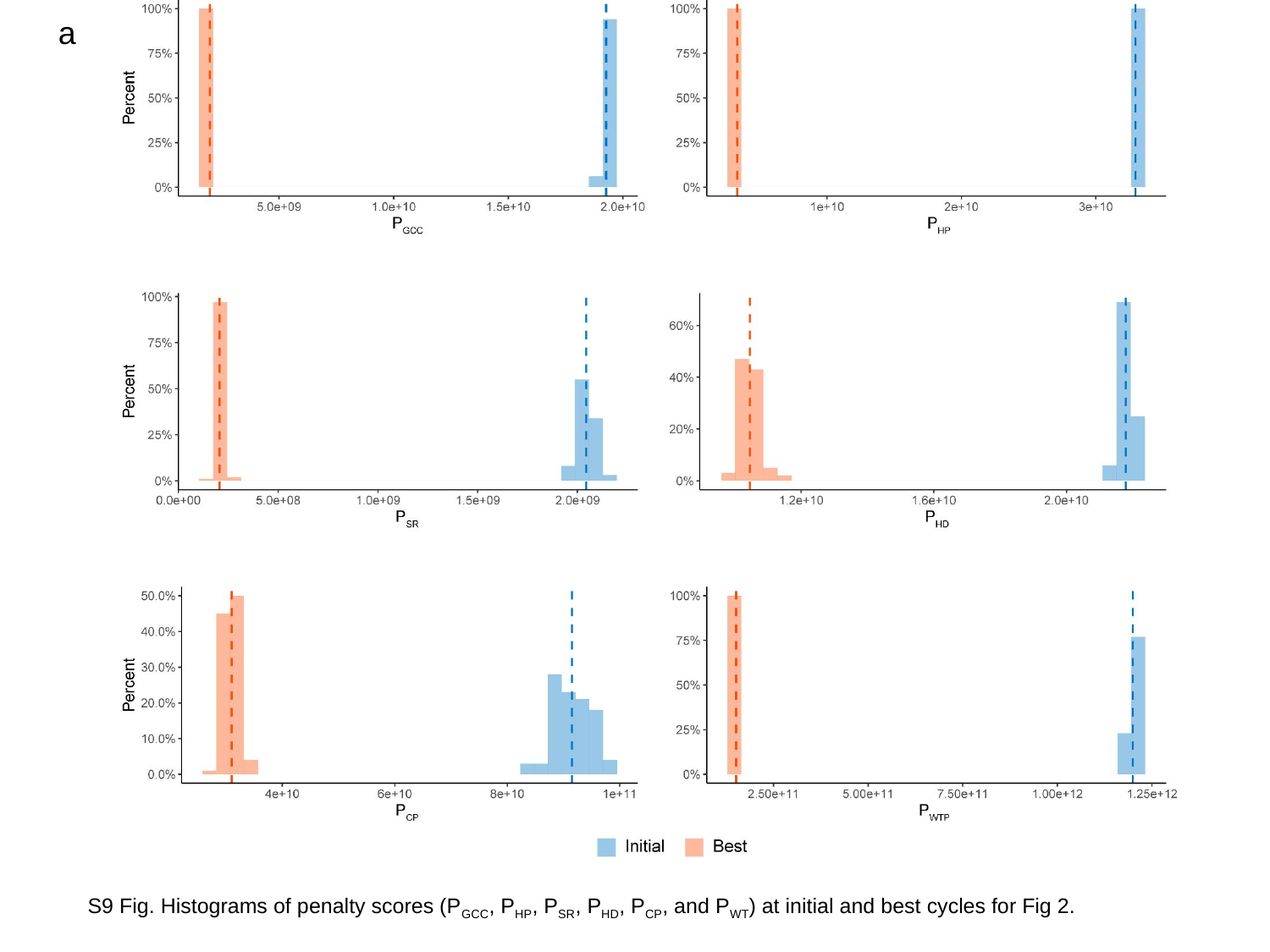

a
S9 Fig. Histograms of penalty scores (PGCC, PHP, PSR, PHD, PCP, and PWT) at initial and best cycles for Fig 2.

## Slide 2
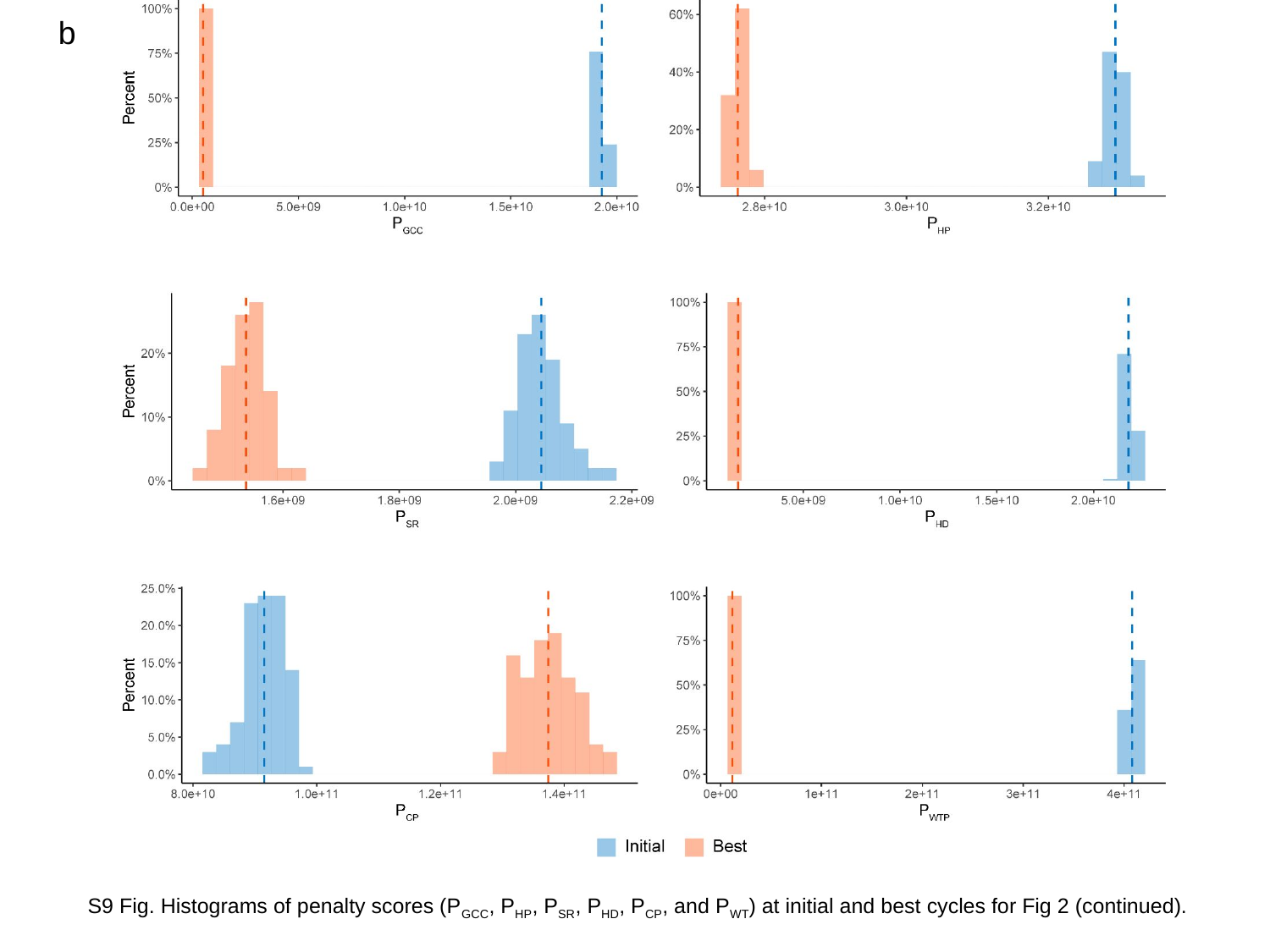

b
S9 Fig. Histograms of penalty scores (PGCC, PHP, PSR, PHD, PCP, and PWT) at initial and best cycles for Fig 2 (continued).

## Slide 3
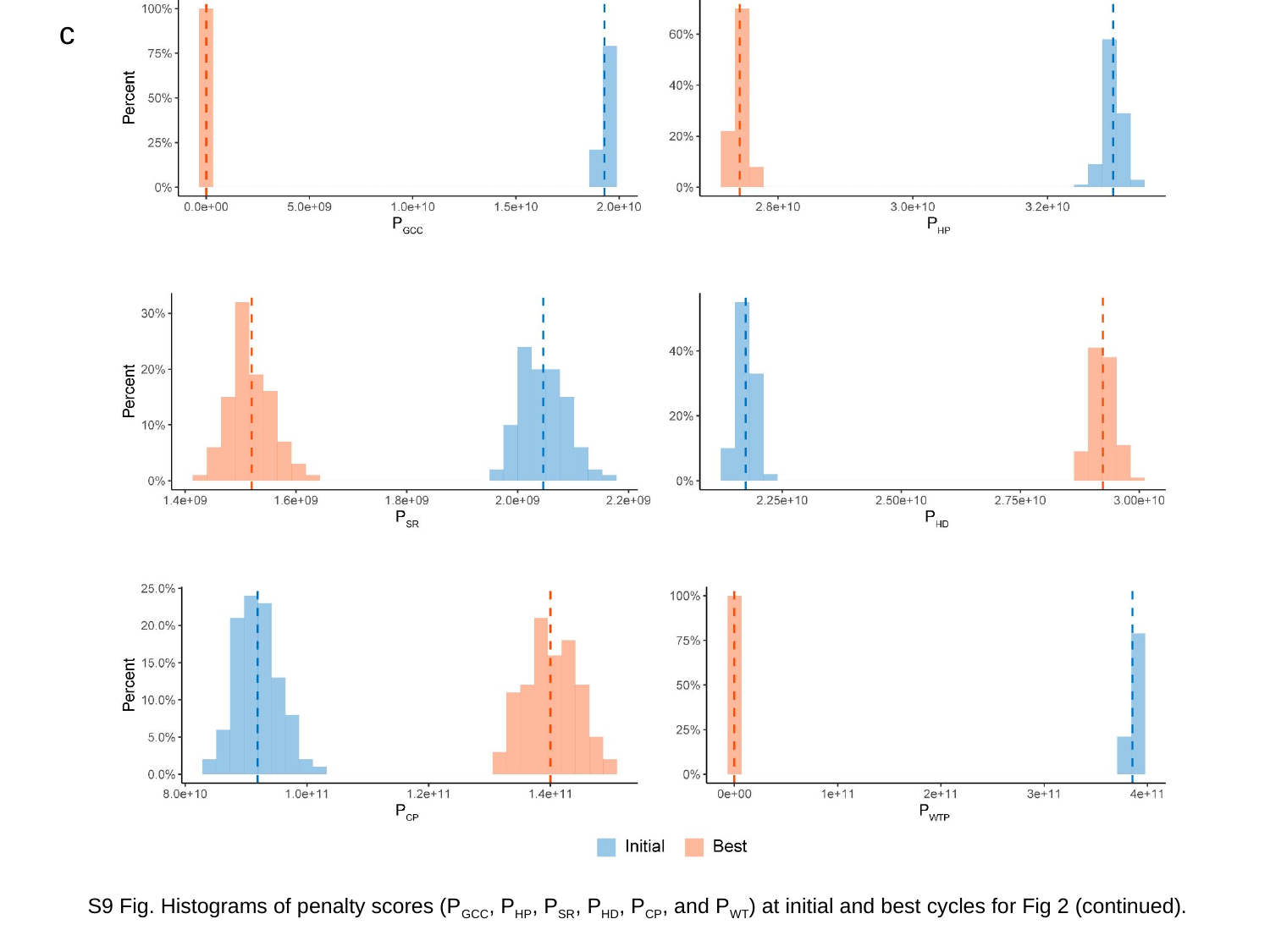

c
S9 Fig. Histograms of penalty scores (PGCC, PHP, PSR, PHD, PCP, and PWT) at initial and best cycles for Fig 2 (continued).

## Slide 4
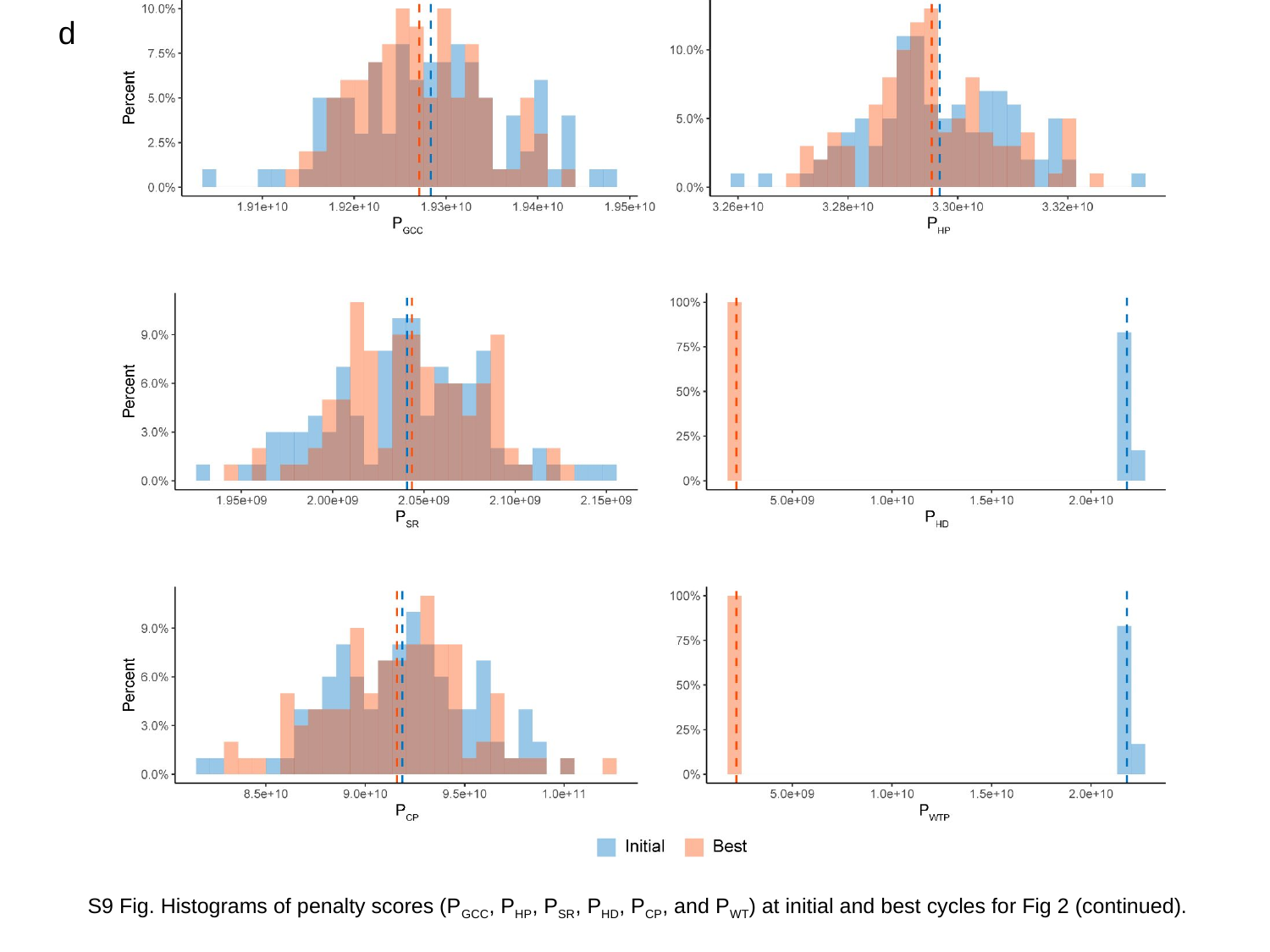

d
S9 Fig. Histograms of penalty scores (PGCC, PHP, PSR, PHD, PCP, and PWT) at initial and best cycles for Fig 2 (continued).
