# Supplementary material for: Development of a program for in silico optimized selection of oligonucleotide-based molecular barcodes
Source: PLoS One. 2021 Feb 18;16(2):e0246354. doi: 10.1371/journal.pone.0246354 (PMC7891705; doi:10.1371/journal.pone.0246354)
Supplement: S10 Fig — (PPTX) [file pone.0246354.s010.pptx]

## Slide 1
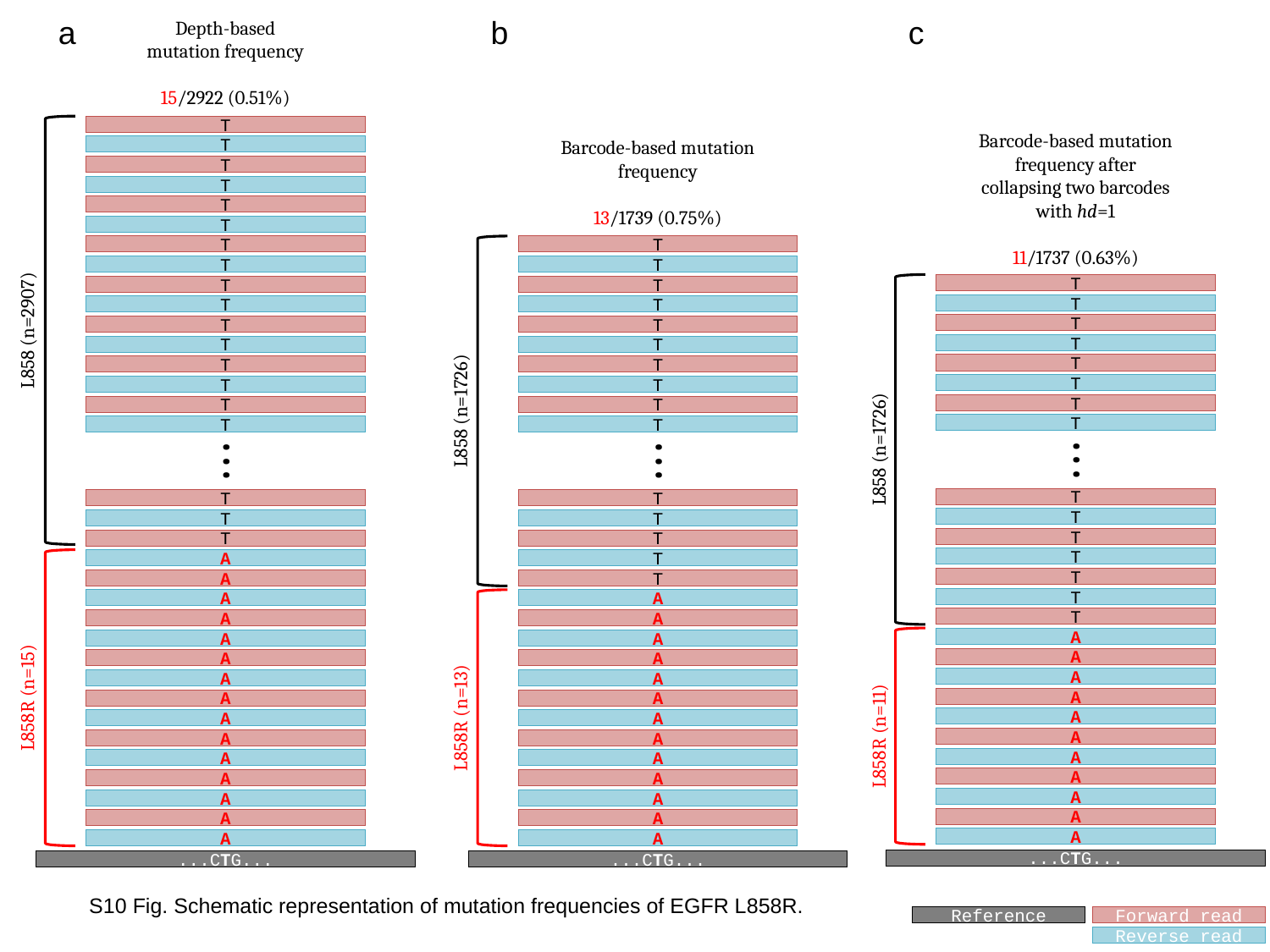

a
b
c
Depth-based mutation frequency
15/2922 (0.51%)
T
Barcode-based mutation frequency after collapsing two barcodes with hd=1
11/1737 (0.63%)
Barcode-based mutation frequency
13/1739 (0.75%)
T
T
T
T
T
T
T
T
T
T
T
T
T
T
T
L858 (n=2907)
T
T
T
T
T
T
T
T
T
T
T
T
L858 (n=1726)
T
T
T
T
T
T
L858 (n=1726)
•••
•••
•••
T
T
T
T
T
T
T
T
T
T
A
T
T
A
T
T
A
A
T
A
A
A
A
A
A
A
A
A
A
A
L858R (n=15)
A
A
A
L858R (n=13)
A
A
A
L858R (n=11)
A
A
A
A
A
A
A
A
A
A
A
A
A
A
A
A
A
A
...CTG...
...CTG...
...CTG...
S10 Fig. Schematic representation of mutation frequencies of EGFR L858R.
Reference
Forward read
Reverse read
