# Supplementary material for: Development of a program for in silico optimized selection of oligonucleotide-based molecular barcodes
Source: PLoS One. 2021 Feb 18;16(2):e0246354. doi: 10.1371/journal.pone.0246354 (PMC7891705; doi:10.1371/journal.pone.0246354)
Supplement: S11 Fig — (PPTX) [file pone.0246354.s011.pptx]

## Slide 1
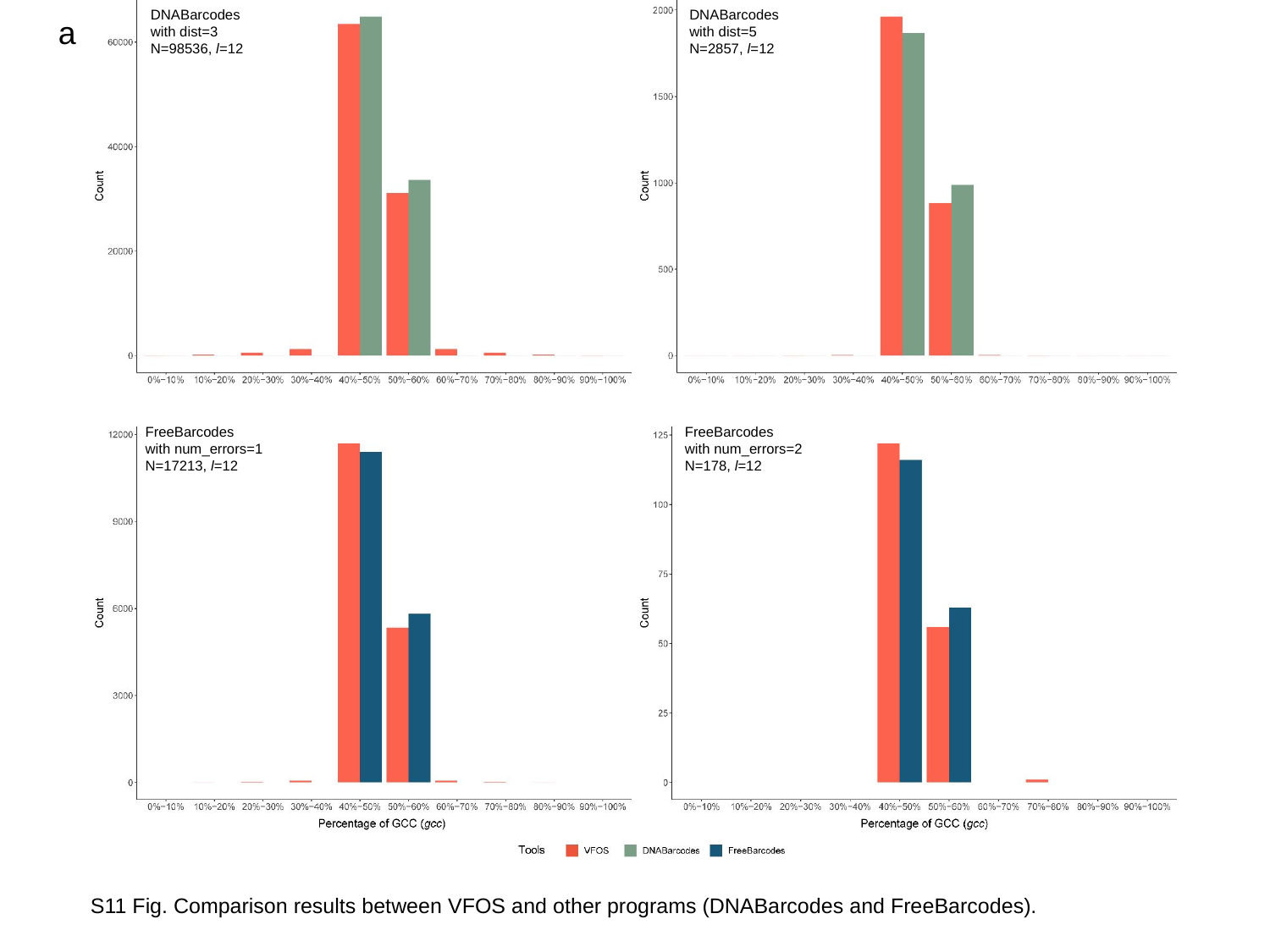

DNABarcodes
with dist=3
N=98536, l=12
DNABarcodes
with dist=5
N=2857, l=12
a
FreeBarcodes
with num_errors=1
N=17213, l=12
FreeBarcodes
with num_errors=2
N=178, l=12
S11 Fig. Comparison results between VFOS and other programs (DNABarcodes and FreeBarcodes).

## Slide 2
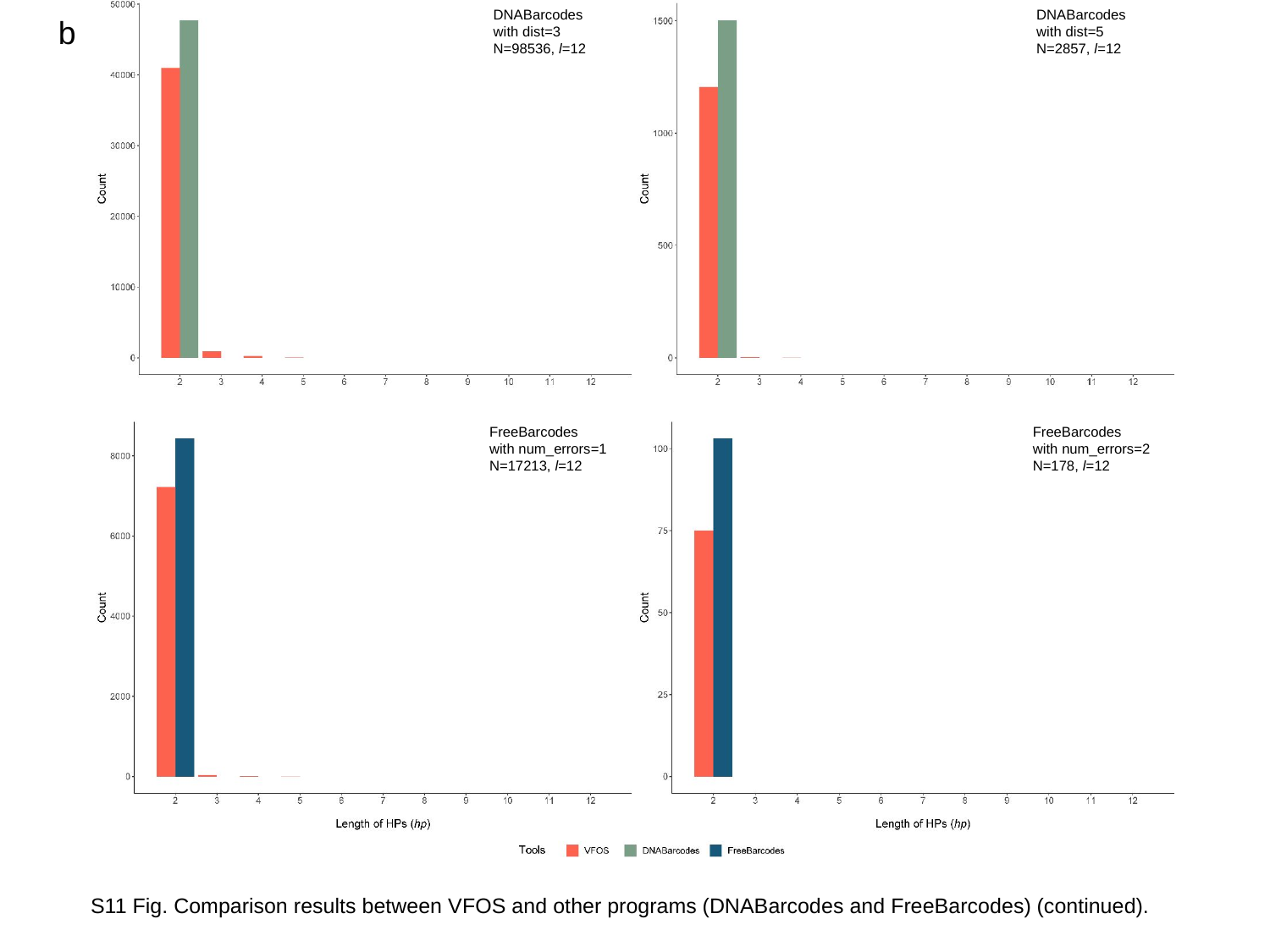

DNABarcodes
with dist=3
N=98536, l=12
DNABarcodes
with dist=5
N=2857, l=12
b
FreeBarcodes
with num_errors=1
N=17213, l=12
FreeBarcodes
with num_errors=2
N=178, l=12
S11 Fig. Comparison results between VFOS and other programs (DNABarcodes and FreeBarcodes) (continued).

## Slide 3
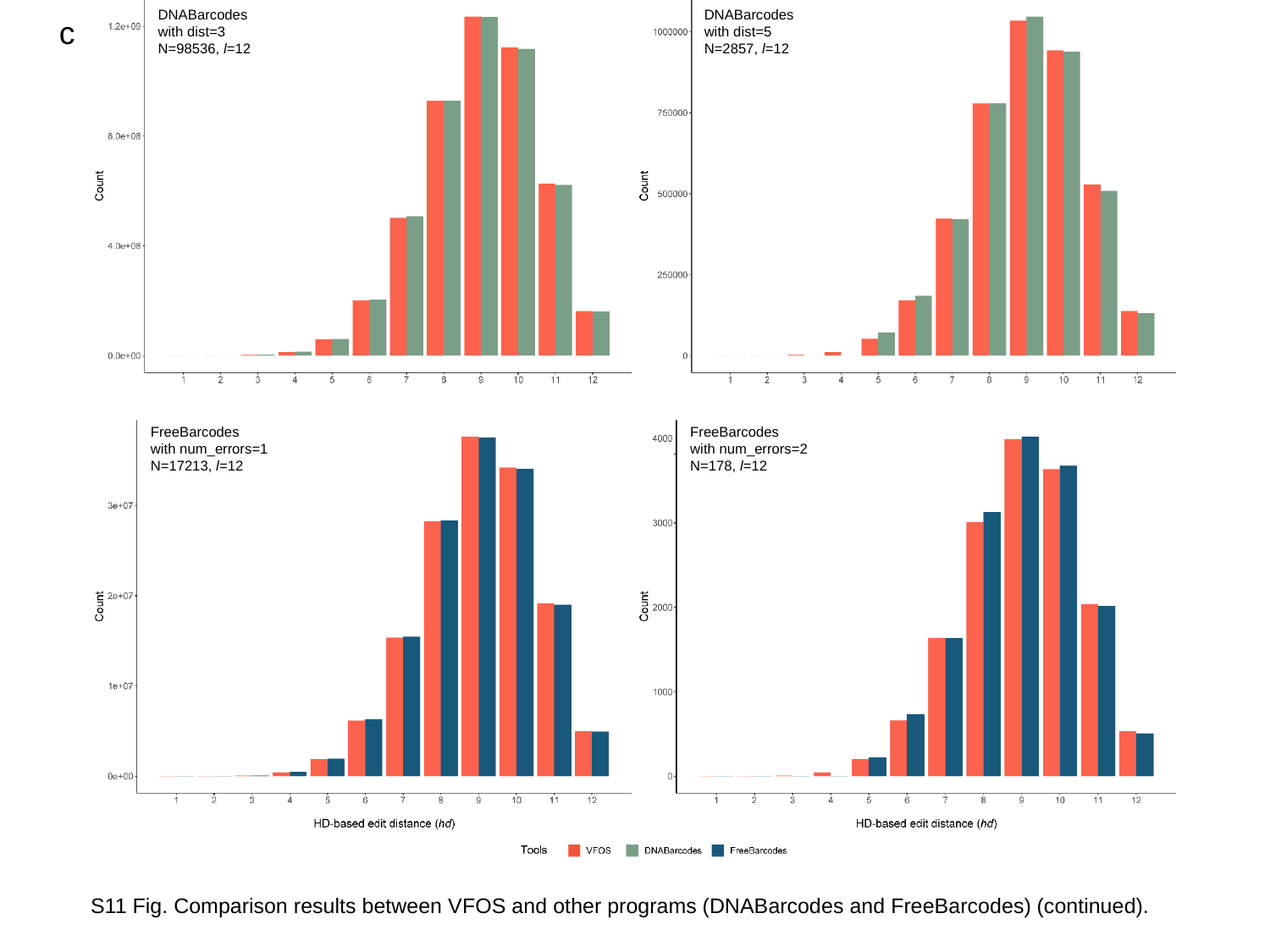

DNABarcodes
with dist=3
N=98536, l=12
DNABarcodes
with dist=5
N=2857, l=12
c
FreeBarcodes
with num_errors=1
N=17213, l=12
FreeBarcodes
with num_errors=2
N=178, l=12
S11 Fig. Comparison results between VFOS and other programs (DNABarcodes and FreeBarcodes) (continued).

## Slide 4
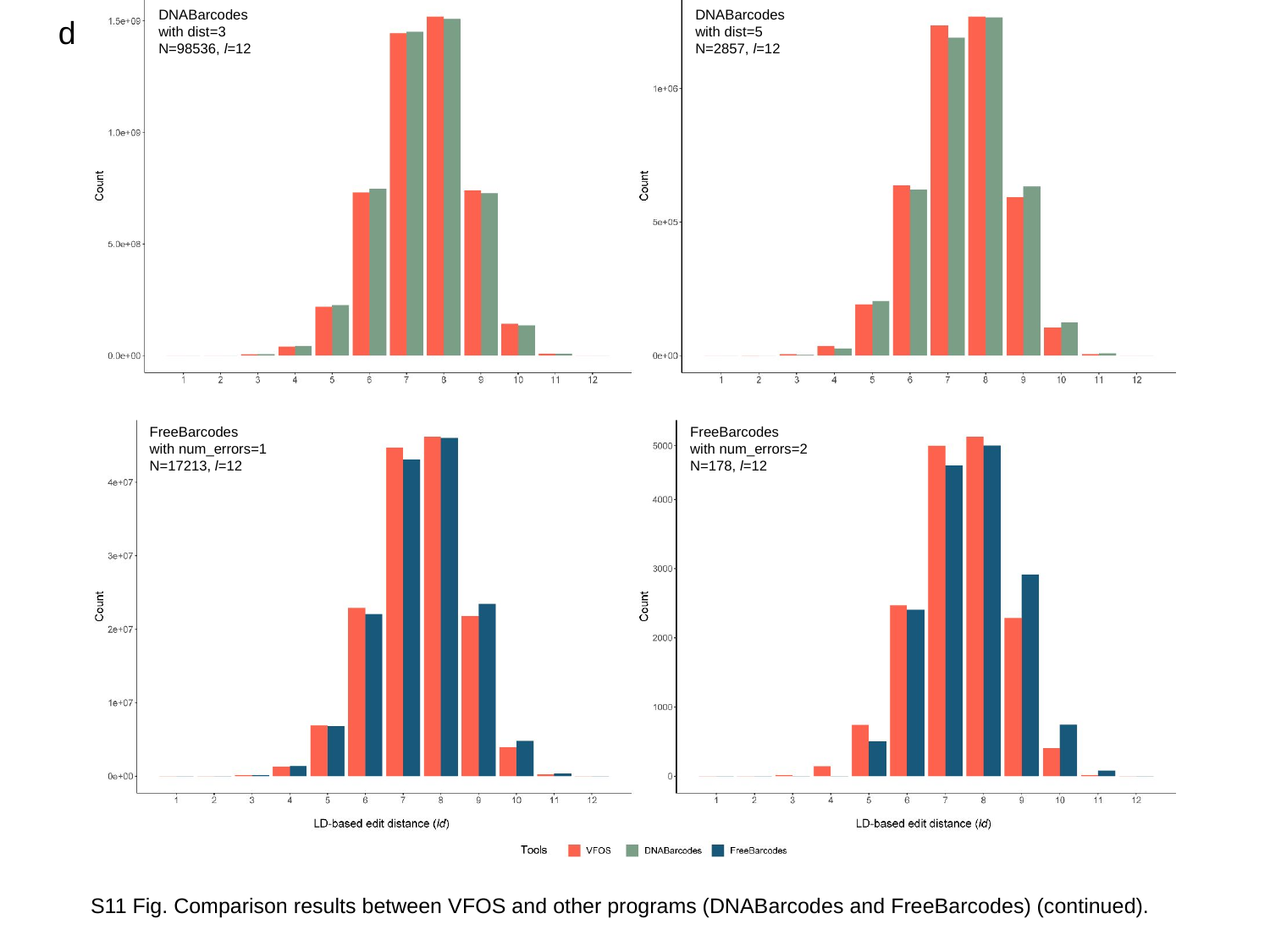

DNABarcodes
with dist=3
N=98536, l=12
DNABarcodes
with dist=5
N=2857, l=12
d
FreeBarcodes
with num_errors=1
N=17213, l=12
FreeBarcodes
with num_errors=2
N=178, l=12
S11 Fig. Comparison results between VFOS and other programs (DNABarcodes and FreeBarcodes) (continued).

## Slide 5
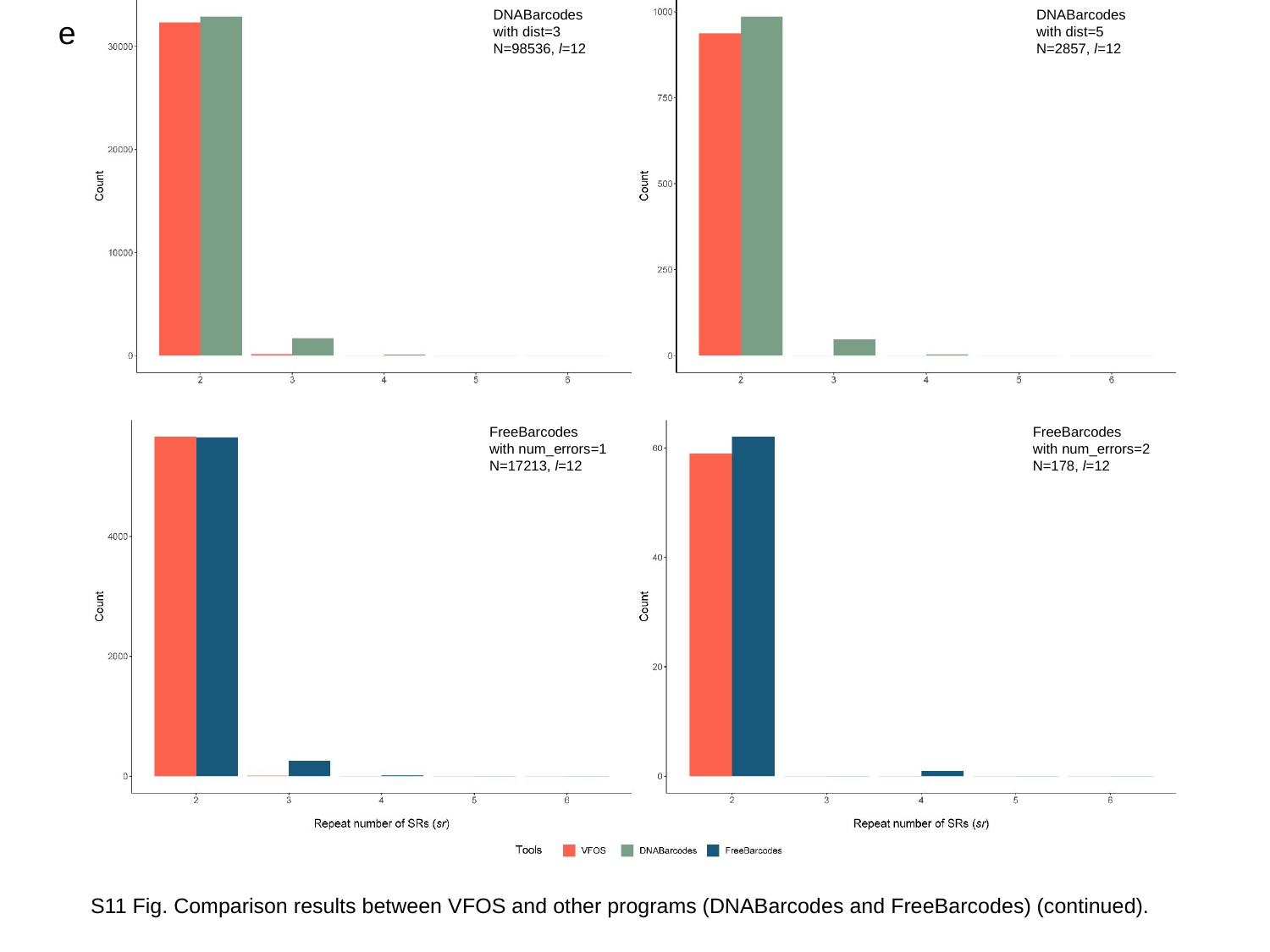

DNABarcodes
with dist=3
N=98536, l=12
DNABarcodes
with dist=5
N=2857, l=12
e
FreeBarcodes
with num_errors=1
N=17213, l=12
FreeBarcodes
with num_errors=2
N=178, l=12
S11 Fig. Comparison results between VFOS and other programs (DNABarcodes and FreeBarcodes) (continued).
